# Supplementary material for: A Multivariable Mendelian Randomization Study of Systolic and Diastolic Blood Pressure, Lipid Profile, and Heart Failure Subtypes
Source: Genes (Basel). 2024 Aug 27;15(9):1126. doi: 10.3390/genes15091126 (PMC11431108; doi:10.3390/genes15091126)
Supplement: Supplementary file 1 [file genes-15-01126-s001.zip › genes-3101763-supplementary.pdf]

Table S1. Summary of Genome-wide Association Studies on Blood Pressure and Lipids in the UK Biobank.

Trait	Sample Size	Independent GWS Loci
**SBP**	315180	171
**DBP**	315180	152
**PP**	315180	232
**HDL-C**	294484	247
**LDL-C**	321090	224
**Triglyceride**	321433	227

SBP: Systolic Blood Pressure; DBP: Diastolic Blood Pressure; PP: Pulse Pressure; HDL-C: High-density Lipoprotein Cholesterol; LDL-C: Low-density Lipoprotein Cholesterol; GWS: Genome-wide Significant.

Table S2. GWAS Summary Used for Mendelian Randomization Analysis of Systolic Blood Pressure (SBP).

rsID	Chr	Position (hg19)	Gene	EA	NEA	SBP				HFrEF				HFpEF			
						EAF	Beta (95% CI)	P	N	EAF	OR (95% CI)	P	N	EAF	OR (95% CI)	P	N
rs2493291	1	3329384	PRDM16	T	C	0.14	0.48 (0.34, 0.61)	1.30E-12	315180	0.14	1.03 (1.01, 1.06)	0.016	395066	0.14	1 (0.97, 1.02)	0.75	394846
rs34071855	1	10798489	CASZ1	G	C	0.34	0.31 (0.22, 0.41)	2.00E-10	315180	0.34	1.03 (1.01, 1.05)	0.0047	395066	0.34	1.02 (1, 1.04)	0.02	394846
rs10449752	1	56617947	-	G	T	0.38	0.27 (0.17, 0.36)	2.70E-08	315180	0.38	1.02 (1, 1.04)	0.034	395066	0.38	0.99 (0.98, 1.01)	0.48	394846
rs7553325	1	67010104	SGIP1	T	C	0.49	0.26 (0.17, 0.35)	1.20E-08	315180	0.49	1 (0.98, 1.02)	0.9	395066	0.49	0.98 (0.97, 1)	0.076	394846
rs3790604	1	1.13E+08	WNT2B	A	C	0.07	0.61 (0.44, 0.78)	5.40E-12	315180	0.07	1.04 (1.01, 1.08)	0.012	395066	0.07	1.03 (1, 1.07)	0.073	394846
rs3827750	1	2.31E+08	AGT	T	C	0.11	0.46 (0.31, 0.6)	6.40E-10	315180	0.11	1.02 (1, 1.05)	0.09	410803	0.11	1.01 (0.98, 1.04)	0.55	410583
rs12714414	2	651407	-	T	C	0.85	0.39 (0.26, 0.52)	6.70E-09	315180	0.83	1.06 (1.04, 1.09)	5.50E-07	410810	0.83	1.04 (1.01, 1.06)	0.0025	410590
rs116734066	2	25262928	EFR3B	C	T	0.91	0.48 (0.32, 0.64)	2.00E-09	315180	0.91	0.99 (0.96, 1.02)	0.52	395066	0.91	1.03 (1, 1.06)	0.077	394846
rs1275988	2	26914364	KCNK3	C	T	0.38	0.4 (0.31, 0.49)	4.30E-17	315180	0.39	1.01 (0.99, 1.03)	0.34	410681	0.39	1.02 (1, 1.03)	0.078	410461
rs981037	2	56194773	-	C	T	0.42	0.27 (0.18, 0.36)	6.60E-09	315180	0.43	1.01 (0.99, 1.03)	0.19	410800	0.43	1.01 (0.99, 1.02)	0.47	410580
rs1010239	2	86451502	REEP1	C	T	0.65	0.27 (0.17, 0.36)	4.50E-08	315180	0.66	1.02 (1, 1.04)	0.086	410810	0.66	0.99 (0.98, 1.01)	0.51	410590
rs112641450	2	1.14E+08	RABL2A	G	A	0.82	0.34 (0.22, 0.46)	1.00E-08	315180	0.83	0.98 (0.95, 1)	0.04	395066	0.83	1 (0.98, 1.03)	0.93	394846
rs12694966	2	1.59E+08	PKP4	G	A	0.64	0.26 (0.17, 0.36)	4.50E-08	315180	0.63	1.02 (1, 1.03)	0.1	410758	0.63	1 (0.98, 1.02)	0.99	410538
rs268263	2	1.65E+08	-	A	T	0.75	0.53 (0.43, 0.64)	6.40E-23	315180	0.73	0.98 (0.96, 1)	0.067	395066	0.73	0.99 (0.97, 1.01)	0.24	394846
rs1250258	2	2.16E+08	FN1	C	T	0.26	0.36 (0.26, 0.47)	4.60E-12	315180	0.26	1.06 (0.97, 1.16)	0.22	15729	0.26	0.94 (0.87, 1.02)	0.15	15729
rs904475	3	11535417	ATG7	A	G	0.63	0.29 (0.2, 0.39)	7.80E-10	315180	0.62	1 (0.99, 1.02)	0.66	395066	0.62	1.02 (1, 1.04)	0.068	394846
rs113913938	3	41868154	ULK4	G	A	0.87	0.47 (0.33, 0.61)	6.50E-11	315180	0.87	0.98 (0.95, 1)	0.095	395066	0.87	0.99 (0.97, 1.02)	0.72	394846
rs35593046	3	53553923	CACNA1D	G	T	0.73	0.29 (0.19, 0.39)	3.60E-08	315180	0.75	1.04 (1.02, 1.06)	0.00032	395066	0.75	1.02 (1, 1.04)	0.12	394846
rs1529427	3	1.34E+08	AMOTL2	C	T	0.65	0.28 (0.18, 0.37)	8.90E-09	315180	0.65	0.99 (0.97, 1.01)	0.33	395066	0.65	1 (0.99, 1.02)	0.65	394846
rs11721984	4	38343935	-	C	T	0.54	0.26 (0.17, 0.35)	3.00E-08	315180	0.55	1.01 (0.99, 1.03)	0.37	395066	0.55	1.02 (1, 1.04)	0.033	394846
rs77514763	4	77404635	SHROOM3	G	A	0.34	0.27 (0.17, 0.36)	3.80E-08	315180	0.35	1.02 (1, 1.04)	0.038	410752	0.35	1.01 (0.99, 1.03)	0.23	410532
rs10029530	4	1.56E+08	-	A	T	0.34	0.33 (0.24, 0.43)	8.20E-12	315180	0.34	1.05 (0.97, 1.15)	0.23	15745	0.34	1.01 (0.93, 1.09)	0.85	15745
rs13154549	5	61944322	IPO11	G	A	0.07	0.5 (0.32, 0.69)	4.30E-08	315180	0.07	1.02 (0.99, 1.06)	0.18	395066	0.07	1.02 (0.99, 1.06)	0.19	394846
rs6452769	5	87389027	TMEM161B	G	A	0.8	0.34 (0.22, 0.45)	7.20E-09	315180	0.78	1 (0.97, 1.02)	0.68	395066	0.78	1 (0.97, 1.02)	0.67	394846
rs6595838	5	1.28E+08	FBN2	A	G	0.31	0.32 (0.23, 0.42)	1.20E-10	315180	0.29	1.03 (1.01, 1.05)	0.0021	395066	0.29	1 (0.98, 1.02)	0.69	394846
rs13436194	5	1.58E+08	-	A	G	0.57	0.32 (0.22, 0.41)	1.30E-11	315180	0.56	0.99 (0.91, 1.07)	0.77	15746	0.56	0.97 (0.9, 1.04)	0.38	15746
rs12197514	6	27553784	ZNF184	T	C	0.65	0.28 (0.19, 0.38)	6.10E-09	315180	0.64	1 (0.98, 1.02)	0.87	395066	0.64	1.01 (1, 1.03)	0.11	394846
rs2971608	6	97039757	FHL5	C	T	0.22	0.35 (0.24, 0.46)	3.40E-10	315180	0.23	1 (0.91, 1.11)	0.94	15651	0.23	1.01 (0.93, 1.1)	0.82	15651
rs3752848	6	1.18E+08	GOPC	C	T	0.51	0.28 (0.19, 0.37)	7.90E-10	315180	0.51	1 (0.98, 1.02)	0.8	410701	0.51	1 (0.98, 1.01)	0.59	410481
rs17082033	6	1.52E+08	ESR1	T	A	0.1	0.41 (0.27, 0.56)	4.80E-08	315180	0.1	1.03 (1, 1.06)	0.087	410672	0.1	1.03 (1, 1.06)	0.075	410452

rs9294987	6	1.7E+08	-	C	T	0.5	0.26 (0.17, 0.35)	1.70E-08	315180	0.5	0.99 (0.97, 1)	0.15	395066	0.5	0.99 (0.97, 1.01)	0.31	394846
rs115525024	7	27236559	HOXA13	G	T	0.93	0.66 (0.48, 0.83)	1.60E-13	315180	0.91	1.05 (1.01, 1.08)	0.0057	410803	0.91	1.02 (0.99, 1.05)	0.21	410583
rs199696982	7	45987430	IGFBP3	G	C	0.55	0.34 (0.25, 0.43)	3.70E-13	315180	0.56	0.99 (0.97, 1)	0.15	395066	0.56	1.01 (0.99, 1.03)	0.22	394846
rs10269774	7	92253972	CDK6	G	A	0.68	0.43 (0.33, 0.52)	4.10E-18	315180	0.66	1.04 (1.02, 1.06)	9.10E-05	410811	0.66	1.02 (1.01, 1.04)	0.0099	410591
rs1813742	7	1.31E+08	MKLN1	T	C	0.59	0.33 (0.24, 0.42)	1.70E-12	315180	0.57	1 (0.99, 1.02)	0.74	395066	0.57	1 (0.98, 1.02)	0.84	394846
rs17173238	7	1.51E+08	PRKAG2	G	A	0.29	0.3 (0.2, 0.4)	3.40E-09	315180	0.29	1.01 (0.99, 1.03)	0.23	410747	0.29	1.02 (1, 1.04)	0.038	410527
rs2911968	8	6488505	MCPH1	C	T	0.38	0.27 (0.17, 0.36)	2.50E-08	315180	0.39	0.99 (0.97, 1.01)	0.22	410725	0.39	1.01 (0.99, 1.03)	0.5	410505
rs4512422	8	10258889	MSRA	C	T	0.44	0.38 (0.28, 0.47)	7.90E-16	315180	0.46	0.99 (0.98, 1.01)	0.48	395066	0.46	1.02 (1, 1.04)	0.024	394846
rs73563812	8	25900405	EBF2	G	T	0.76	0.33 (0.23, 0.44)	9.70E-10	315180	0.77	1 (0.98, 1.03)	0.66	395066	0.77	1.01 (0.98, 1.03)	0.6	394846
rs72649639	8	51891208	SNTG1	G	A	0.17	0.37 (0.25, 0.49)	1.00E-09	315180	0.18	0.99 (0.97, 1.01)	0.44	410743	0.18	1 (0.97, 1.02)	0.76	410523
rs74900445	8	95268903	GEM	C	T	0.12	0.4 (0.26, 0.54)	1.50E-08	315180	0.12	1.02 (1, 1.05)	0.11	410777	0.12	1.02 (0.99, 1.05)	0.15	410557
rs10096038	8	1.41E+08	TRAPPC9	C	T	0.29	0.32 (0.22, 0.42)	3.10E-10	315180	0.3	0.98 (0.96, 1)	0.051	395066	0.3	1.01 (0.99, 1.03)	0.19	394846
rs10817007	9	1.13E+08	SVEP1	G	T	0.13	0.41 (0.27, 0.54)	4.40E-09	315180	0.13	1.02 (1, 1.05)	0.1	410801	0.13	1.01 (0.98, 1.04)	0.42	410581
rs11527181	10	64546164	ADO	A	G	0.57	0.32 (0.23, 0.41)	7.40E-12	315180	0.58	0.99 (0.97, 1.01)	0.44	395066	0.58	0.99 (0.97, 1.01)	0.2	394846
rs4746139	10	75407649	SYNPO2L	C	A	0.14	0.45 (0.32, 0.58)	2.50E-11	315180	0.14	0.93 (0.91, 0.95)	4.80E-09	410798	0.14	0.96 (0.94, 0.98)	0.0015	410578
rs117472357	10	89614816	KLLN	T	C	0.04	0.66 (0.43, 0.89)	2.20E-08	315180	0.04	1 (0.96, 1.05)	0.9	410809	0.04	1.05 (1, 1.1)	0.056	410589
rs56092448	10	1.34E+08	INPP5A	T	C	0.11	0.41 (0.26, 0.55)	2.10E-08	315180	0.11	0.99 (0.96, 1.01)	0.32	410807	0.11	1 (0.98, 1.03)	0.83	410587
rs4980379	11	1888614	LSP1	T	C	0.36	0.42 (0.33, 0.52)	1.60E-18	315180	0.36	1.04 (1.03, 1.06)	2.90E-06	410805	0.36	1.02 (1, 1.03)	0.088	410585
rs4442541	11	10669172	MRVI1	A	G	0.31	0.29 (0.19, 0.39)	4.80E-09	315180	0.32	0.95 (0.87, 1.04)	0.23	15714	0.32	0.96 (0.89, 1.03)	0.25	15714
rs11525445	11	55351007	OR4C16	C	T	0.81	0.34 (0.22, 0.45)	7.70E-09	315180	0.82	1.01 (0.99, 1.03)	0.39	410729	0.82	1 (0.97, 1.02)	0.72	410509
rs4936098	11	1.3E+08	ADAMTS8	A	G	0.38	0.4 (0.3, 0.49)	1.40E-16	315180	0.38	0.99 (0.97, 1.01)	0.33	395066	0.38	1 (0.98, 1.02)	0.84	394846
rs73437338	12	90054619	ATP2B1	T	C	0.83	0.62 (0.5, 0.74)	2.70E-23	315180	0.84	0.95 (0.93, 0.98)	0.0001	410811	0.84	1.02 (0.99, 1.04)	0.2	410591
rs35427	12	1.16E+08	-	T	G	0.62	0.29 (0.2, 0.39)	1.60E-09	315180	0.61	1.01 (0.99, 1.03)	0.17	395066	0.61	1.04 (1.02, 1.05)	0.00028	394846
rs138110118	13	27904650	RASL11A	T	G	0.89	0.46 (0.32, 0.6)	3.80E-10	315180	0.89	1 (0.97, 1.03)	0.92	410720	0.89	1 (0.97, 1.02)	0.72	410500
rs11616710	13	1.15E+08	UPF3A	T	C	0.1	0.49 (0.33, 0.65)	1.50E-09	315180	0.1	0.98 (0.95, 1.01)	0.29	395066	0.1	1.03 (1, 1.06)	0.074	394846
rs150170911	14	73513400	RBM25	T	C	0.09	0.49 (0.33, 0.65)	1.80E-09	315180	0.09	0.99 (0.96, 1.02)	0.54	410786	0.09	1 (0.97, 1.03)	0.76	410566
rs28929474	14	94844947	SERPINA1	C	T	0.98	0.89 (0.57, 1.21)	4.10E-08	315180	0.98	1.01 (0.95, 1.08)	0.74	395066	0.98	1.01 (0.95, 1.08)	0.78	394846
rs75989961	14	98522675	-	G	T	0.08	0.52 (0.35, 0.68)	8.20E-10	315180	0.08	1 (0.97, 1.03)	0.85	395066	0.08	1 (0.97, 1.03)	0.99	394846
rs12910173	15	42266428	EHD4	A	G	0.21	0.32 (0.2, 0.43)	4.50E-08	315180	0.21	1.04 (1.02, 1.06)	0.00077	395066	0.21	1 (0.98, 1.03)	0.85	394846
rs55868854	15	75035503	CYP1A2	A	C	0.27	0.36 (0.26, 0.46)	3.10E-12	315180	0.29	1.04 (1.02, 1.06)	7.80E-05	410807	0.29	0.99 (0.97, 1.01)	0.24	410587
rs3211995	16	2089006	SLC9A3R2	G	A	0.84	0.39 (0.26, 0.51)	1.00E-09	315180	0.83	1.01 (0.99, 1.04)	0.24	395066	0.83	1 (0.97, 1.02)	0.87	394846
rs148753960	16	31047822	STX4	C	T	0.99	1.33 (0.85, 1.8)	4.10E-08	315180	0.99	0.95 (0.87, 1.04)	0.3	395066	-	-	-	-
rs61657590	16	65266649	-	C	T	0.51	0.28 (0.19, 0.37)	2.90E-09	315180	0.51	1 (0.98, 1.02)	0.98	395066	0.51	0.99 (0.97, 1.01)	0.19	394846
rs10852858	17	1357751	CRK	T	C	0.44	0.26 (0.17, 0.36)	1.50E-08	315180	0.44	1.02 (1, 1.03)	0.11	395066	0.44	1.01 (0.99, 1.03)	0.37	394846

rs4480845	17	1958609	HIC1	T	C	0.36	0.39 (0.29, 0.48)	1.20E-15	315180	0.38	0.98 (0.96, 1)	0.079	395066	0.38	0.99 (0.98, 1.01)	0.53	394846
rs408315	17	7106475	DLG4	A	G	0.97	0.84 (0.56, 1.13)	6.70E-09	315180	0.97	0.97 (0.92, 1.03)	0.31	395066	0.97	0.99 (0.94, 1.05)	0.71	394846
rs1468270	17	46125191	NFE2L1	C	T	0.8	0.36 (0.25, 0.47)	5.10E-10	315180	0.79	1.01 (0.99, 1.03)	0.41	410811	0.79	0.99 (0.97, 1.01)	0.46	410591
rs3786132	17	60768921	MRC2	C	A	0.43	0.3 (0.21, 0.39)	8.20E-11	315180	0.41	1.01 (0.99, 1.03)	0.39	410763	0.41	1.01 (1, 1.03)	0.13	410543
rs6504213	17	62381714	-	C	T	0.59	0.28 (0.19, 0.37)	2.00E-09	315180	0.57	1.01 (0.99, 1.03)	0.34	395066	0.57	1.01 (0.99, 1.03)	0.5	394846
rs2306526	17	76798362	USP36	C	T	0.48	0.28 (0.19, 0.37)	1.50E-09	315180	0.47	1.03 (1.01, 1.05)	0.00068	410686	0.47	1.02 (1, 1.04)	0.035	410466
rs1397985	18	42073305	-	G	T	0.68	0.3 (0.21, 0.4)	6.60E-10	315180	0.67	1.01 (0.99, 1.03)	0.45	410763	0.67	1.01 (0.99, 1.03)	0.44	410543
rs11874246	18	42596789	-	T	C	0.29	0.29 (0.19, 0.39)	1.20E-08	315180	0.28	1 (0.98, 1.02)	0.99	410732	0.28	0.98 (0.96, 1)	0.034	410512
rs55678414	19	2177625	DOT1L	G	T	0.06	0.63 (0.43, 0.82)	2.40E-10	315180	0.07	0.95 (0.92, 0.99)	0.0076	410695	0.07	1.01 (0.97, 1.05)	0.57	410475
rs2613765	19	5066330	KDM4B	G	A	0.53	0.26 (0.17, 0.35)	2.70E-08	315180	0.55	1.02 (1, 1.04)	0.07	395066	0.55	1.02 (1, 1.04)	0.036	394846
rs1887320	20	10965998	JAG1	A	G	0.48	0.32 (0.23, 0.41)	4.40E-12	315180	0.47	1 (0.99, 1.02)	0.58	410812	0.47	0.99 (0.97, 1)	0.12	410592

Chr: Chromosome; EA: Effect Allele; NEA: Non-effect Allele; EAF: Effect Allele Frequency.

SNPs excluded in the MR-PRESSO analysis of HFrEF: rs12714414, rs4746139, rs4980379, rs73437338.

SNPs excluded in the MR-PRESSO analysis of HFpEF: rs4746139.

Table S3. GWAS Summary Used for Mendelian Randomization Analysis of Diastolic Blood Pressure (DBP).

rsID	Chr	Position (hg19)	Gene	EA	NEA	DBP				HFrEF				HFpEF			
						EAF	Beta (95% CI)	P	N	EAF	OR (95% CI)	P	N	EAF	OR (95% CI)	P	N
rs2493288	1	3330884	PRDM16	A	G	0.14	0.3 (0.22, 0.37)	5.20E-15	315186	0.15	1.03 (1.01, 1.06)	0.019	395066	0.15	1 (0.97, 1.02)	0.86	394846
rs55857306	1	11895795	CLCN6	G	A	0.84	0.37 (0.3, 0.44)	1.10E-24	315186	0.84	1.02 (0.99, 1.04)	0.17	410786	0.84	0.98 (0.95, 1)	0.04	410566
rs72654647	1	25022314	CLIC4	A	G	0.25	0.17 (0.11, 0.23)	1.70E-08	315186	0.24	1 (0.98, 1.03)	0.68	395066	0.24	0.99 (0.97, 1.01)	0.48	394846
rs35023236	1	113085452	ST7L	G	C	0.21	0.22 (0.16, 0.29)	9.50E-12	315186	0.2	1.02 (1, 1.04)	0.1	410760	0.2	1 (0.98, 1.02)	0.94	410540
rs882624	1	201735913	NAV1	C	T	0.67	0.19 (0.13, 0.24)	2.40E-11	315186	0.68	1.01 (0.99, 1.03)	0.41	395066	0.68	1 (0.98, 1.02)	0.79	394846
rs6426577	1	227355918	CDC42BPA	C	A	0.43	0.15 (0.1, 0.2)	1.90E-08	315186	0.43	0.99 (0.98, 1.01)	0.42	410763	0.43	1 (0.98, 1.01)	0.74	410543
rs2004776	1	230848702	AGT	T	C	0.24	0.21 (0.15, 0.27)	4.00E-12	315186	0.24	1.02 (1, 1.04)	0.022	410759	0.24	1.01 (0.99, 1.03)	0.51	410539
rs10926985	1	243463189	SDCCAG8	T	C	0.32	0.25 (0.19, 0.3)	1.90E-18	315186	0.32	1.01 (0.99, 1.03)	0.48	395066	0.32	1.01 (0.99, 1.03)	0.41	394846
rs1275977	2	26922855	KCNK3	G	A	0.34	0.2 (0.15, 0.25)	7.30E-13	315186	0.35	1.01 (0.99, 1.03)	0.42	395066	0.35	1.02 (1, 1.04)	0.017	394846
rs6708660	2	43752382	THADA	T	C	0.6	0.16 (0.11, 0.21)	2.30E-09	315186	0.61	1.01 (0.99, 1.03)	0.23	395066	0.61	0.99 (0.97, 1.01)	0.35	394846
rs11677058	2	55097549	EML6	A	G	0.53	0.15 (0.1, 0.2)	2.60E-08	315186	0.52	1 (0.98, 1.02)	0.97	395066	0.52	1 (0.98, 1.01)	0.71	394846
rs9309473	2	73743982	ALMS1	G	A	0.23	0.17 (0.11, 0.23)	4.20E-08	315186	0.23	1.02 (1, 1.04)	0.11	410814	0.23	1 (0.98, 1.02)	0.85	410594
rs72854462	2	145720139	-	G	A	0.25	0.25 (0.19, 0.31)	1.10E-16	315186	0.23	0.97 (0.95, 0.99)	0.0037	410799	0.23	0.98 (0.96, 1)	0.077	410579
rs1446468	2	164963486	-	C	T	0.55	0.16 (0.11, 0.21)	2.00E-09	315186	0.52	0.99 (0.98, 1.01)	0.46	410795	0.52	0.99 (0.97, 1.01)	0.24	410575
rs142556838	2	179747068	CCDC141	T	C	0.09	0.31 (0.22, 0.4)	1.30E-11	315186	0.09	1 (0.97, 1.04)	0.78	410683	0.09	1.03 (1, 1.07)	0.034	410463
rs12693302	2	183211443	PDE1A	G	A	0.35	0.22 (0.16, 0.27)	2.90E-15	315186	0.35	1.02 (1, 1.03)	0.11	410811	0.35	1 (0.99, 1.02)	0.62	410591
rs11718573	3	7487177	GRM7	C	G	0.5	0.16 (0.11, 0.21)	2.40E-09	315186	0.48	1 (0.99, 1.02)	0.67	395066	0.48	0.98 (0.97, 1)	0.062	394846
rs1687295	3	14889756	FGD5	T	C	0.27	0.18 (0.12, 0.23)	3.10E-09	315186	0.29	1.02 (1, 1.05)	0.019	395066	0.29	1 (0.98, 1.02)	0.8	394846
rs60248638	3	41883692	ULK4	A	G	0.13	0.36 (0.28, 0.44)	8.50E-19	315186	0.13	1.03 (1, 1.06)	0.069	395066	0.13	1.01 (0.99, 1.04)	0.33	394846
rs9834975	3	122117663	FAM162A	T	A	0.44	0.16 (0.1, 0.21)	3.90E-09	315186	0.43	1.01 (0.99, 1.02)	0.51	395066	0.43	0.99 (0.97, 1.01)	0.31	394846
rs76438269	3	141059511	ZBTB38	G	A	0.06	0.3 (0.19, 0.41)	4.30E-08	315186	0.06	0.98 (0.95, 1.02)	0.28	410767	0.06	0.99 (0.96, 1.03)	0.63	410547
rs2195636	3	158352440	GFM1	C	T	0.31	0.18 (0.12, 0.23)	4.50E-10	315186	0.3	0.99 (0.97, 1.01)	0.17	410725	0.3	0.99 (0.98, 1.01)	0.6	410505
rs1527351	4	102084331	PPP3CA	T	C	0.66	0.16 (0.1, 0.21)	1.30E-08	315186	0.67	1 (0.99, 1.02)	0.63	410798	0.67	1.01 (0.99, 1.02)	0.56	410578
rs66887589	4	120509279	PDE5A	C	T	0.47	0.15 (0.1, 0.2)	8.80E-09	315186	0.48	1 (0.98, 1.02)	0.81	395066	0.48	1 (0.98, 1.02)	0.79	394846
rs11731886	4	156659819	GUCY1A3	A	C	0.76	0.18 (0.12, 0.24)	3.90E-09	315186	0.76	1.02 (1, 1.04)	0.072	395066	0.76	1.02 (1, 1.04)	0.069	394846
rs35807464	5	61941653	IPO11	A	G	0.07	0.33 (0.23, 0.44)	4.00E-10	315186	0.07	1.02 (0.99, 1.06)	0.18	395066	0.07	1.02 (0.99, 1.06)	0.23	394846
rs1579036	5	122921103	CSNK1G3	A	G	0.32	0.2 (0.14, 0.25)	4.20E-12	315186	0.32	1 (0.98, 1.02)	0.87	395066	0.32	0.98 (0.96, 1)	0.017	394846
rs17717829	5	158470063	EBF1	T	C	0.55	0.16 (0.11, 0.21)	9.60E-10	315186	0.57	1 (0.98, 1.02)	0.77	395066	0.57	1.01 (0.99, 1.03)	0.39	394846
rs2032451	6	26092170	HFE	T	G	0.15	0.26 (0.19, 0.33)	2.30E-12	315186	0.15	1.01 (0.99, 1.03)	0.41	410808	0.15	1.01 (0.98, 1.03)	0.5	410588
rs13437444	6	28070998	ZNF165	C	T	0.78	0.24 (0.18, 0.31)	3.00E-14	315186	0.81	1.02 (1, 1.05)	0.03	410810	0.81	1 (0.98, 1.02)	0.93	410590

rs9296668	6	51838263	PKHD1	G	A	0.48	0.18 (0.13, 0.23)	1.80E-11	315186	-	-	-	-	0.48	0.96 (0.89, 1.03)	0.25	15676
rs9352694	6	79800818	PHIP	G	A	0.39	0.17 (0.12, 0.23)	2.30E-10	315186	0.39	1.04 (1.02, 1.06)	1.30E-05	395066	0.39	1.01 (0.99, 1.03)	0.17	394846
rs9448894	6	80837253	BCKDHB	T	G	0.47	0.15 (0.1, 0.21)	7.80E-09	315186	0.48	1 (0.98, 1.01)	0.75	410814	0.48	1.01 (0.99, 1.03)	0.29	410594
rs2207231	6	152329884	ESR1	A	G	0.9	0.24 (0.15, 0.32)	4.20E-08	315186	0.9	0.98 (0.95, 1.01)	0.11	410742	0.9	0.97 (0.95, 1)	0.071	410522
rs9689079	6	169587148	THBS2	A	G	0.22	0.19 (0.13, 0.26)	1.20E-09	315186	0.23	0.99 (0.97, 1.01)	0.21	410706	0.23	1 (0.98, 1.02)	0.86	410486
rs343011	7	35488349	-	T	G	0.23	0.21 (0.15, 0.27)	1.60E-11	315186	0.24	1.02 (1, 1.04)	0.05	395066	0.24	0.98 (0.96, 1.01)	0.15	394846
rs3823483	7	131010943	MKLN1	C	T	0.55	0.15 (0.1, 0.2)	3.20E-08	315186	0.53	1.01 (0.99, 1.03)	0.39	395066	0.53	0.99 (0.97, 1.01)	0.37	394846
rs11136373	8	1212030	-	G	C	0.63	0.16 (0.1, 0.21)	1.70E-08	315186	0.62	1.01 (0.99, 1.03)	0.3	395066	0.62	1.01 (0.99, 1.02)	0.56	394846
rs2979666	8	6361229	MCPH1; ANGPT2	C	A	0.51	0.16 (0.1, 0.21)	3.70E-09	315186	0.51	1.02 (1, 1.03)	0.073	395066	0.51	1 (0.98, 1.02)	0.94	394846
rs7837979	8	10198534	MSRA	T	C	0.43	0.22 (0.17, 0.27)	1.50E-16	315186	0.42	0.99 (0.97, 1)	0.15	395066	0.42	1.01 (0.99, 1.03)	0.26	394846
rs60392867	8	23404016	SLC25A37	G	A	0.24	0.17 (0.11, 0.23)	4.20E-08	315186	0.24	1 (0.98, 1.02)	0.96	410678	0.24	1 (0.98, 1.02)	0.98	410458
rs11782995	8	120400847	NOV	G	A	0.75	0.25 (0.19, 0.31)	5.20E-16	315186	0.75	1.02 (1, 1.04)	0.076	395066	0.75	0.99 (0.97, 1.01)	0.44	394846
rs4076877	8	144902714	PUF60	C	T	0.95	0.35 (0.23, 0.47)	6.50E-09	315186	0.95	1.05 (1.01, 1.1)	0.023	395066	0.95	1 (0.96, 1.05)	1	394846
rs7034703	9	14461188	-	G	C	0.58	0.15 (0.1, 0.21)	9.40E-09	315186	0.6	1 (0.98, 1.02)	0.76	395066	0.6	1.02 (1, 1.04)	0.027	394846
rs6271	9	136522274	DBH	C	T	0.93	0.37 (0.27, 0.46)	4.20E-13	315186	0.93	1.03 (1, 1.07)	0.088	395066	0.93	1.03 (1, 1.07)	0.093	394846
rs12379849	9	139519819	-	C	T	0.41	0.17 (0.12, 0.22)	4.10E-10	315186	0.43	0.99 (0.98, 1.01)	0.51	395066	0.43	1.01 (0.99, 1.03)	0.28	394846
9:140205269_C_T	9	140205269	EXD3	C	T	0.66	0.16 (0.1, 0.21)	1.70E-08	315186	0.66	1.01 (1, 1.03)	0.14	395066	0.66	1.01 (0.99, 1.03)	0.29	394846
rs1509966	10	64552607	ADO	G	A	0.5	0.21 (0.16, 0.26)	1.20E-15	315186	0.52	1 (0.98, 1.01)	0.7	410779	0.52	0.99 (0.97, 1.01)	0.24	410559
rs1863243	11	10677373	MRVI1	G	A	0.33	0.19 (0.13, 0.24)	2.10E-11	315186	0.35	0.99 (0.97, 1.01)	0.28	395066	0.35	0.99 (0.97, 1.01)	0.42	394846
rs751984	11	61278246	LRRC10B	T	C	0.88	0.31 (0.23, 0.39)	1.60E-13	315186	0.89	1.02 (0.99, 1.05)	0.28	395066	0.88	0.98 (0.95, 1.01)	0.13	394846
rs11021221	11	95308854	-	T	A	0.83	0.24 (0.17, 0.31)	1.10E-11	315186	0.84	1.02 (1, 1.05)	0.051	395066	0.84	1 (0.97, 1.02)	0.85	394846
rs11212087	11	107097881	-	C	T	0.72	0.16 (0.11, 0.22)	2.50E-08	315186	0.73	1.02 (1, 1.04)	0.089	410813	0.73	1.01 (0.99, 1.03)	0.41	410593
rs7129204	11	122527212	UBASH3B	C	G	0.12	0.25 (0.18, 0.33)	3.50E-10	315186	0.12	1.04 (1.01, 1.06)	0.0076	410714	0.12	1.01 (0.98, 1.04)	0.52	410494
rs1590008	12	90107725	ATP2B1	T	C	0.67	0.19 (0.13, 0.24)	2.20E-11	315186	0.66	0.99 (0.97, 1.01)	0.21	410797	0.66	1.02 (1, 1.04)	0.014	410577
rs35441	12	115553115	-	C	T	0.62	0.17 (0.12, 0.23)	1.30E-10	315186	0.62	1.01 (1, 1.03)	0.1	410686	0.62	1.03 (1.01, 1.05)	0.00076	410466
rs4767332	12	115932272	-	A	C	0.58	0.18 (0.12, 0.23)	6.10E-11	315186	0.55	1 (0.98, 1.01)	0.62	410808	0.56	1.02 (1, 1.03)	0.062	410588
rs9548033	13	38289973	TRPC4	C	T	0.43	0.16 (0.11, 0.21)	4.60E-09	315186	0.42	1.02 (0.93, 1.1)	0.73	15661	0.42	1 (0.93, 1.07)	0.94	15661
rs7338606	13	110900111	COL4A1	T	C	0.22	0.21 (0.14, 0.27)	1.60E-10	315186	0.21	1.03 (1.01, 1.05)	0.0029	410801	0.21	1.02 (1, 1.04)	0.083	410581
rs28680296	15	42121538	PLA2G4B; JMJD7; JMJD7-PLA2G4B	G	C	0.34	0.19 (0.14, 0.25)	3.90E-12	315186	0.34	1.01 (1, 1.03)	0.11	410790	0.34	0.99 (0.97, 1.01)	0.33	410570
rs8036173	15	48820068	FBN1	C	T	0.25	0.18 (0.12, 0.24)	2.40E-09	315186	0.25	1 (0.98, 1.02)	0.93	410735	0.25	1.01 (0.99, 1.03)	0.56	410515
rs1440371	15	66941084	RP11-321F6.1	A	G	0.29	0.16 (0.11, 0.22)	2.80E-08	315186	0.28	1.02 (1, 1.04)	0.1	410814	0.28	1 (0.98, 1.02)	0.97	410594
rs3743111	15	71587373	THSD4	A	G	0.62	0.15 (0.09, 0.2)	4.70E-08	315186	0.6	0.99 (0.97, 1.01)	0.16	410752	0.6	0.99 (0.97, 1)	0.13	410532

rs11636952	15	75114322	LMAN1L	T	C	0.31	0.27 (0.22, 0.33)	1.20E-21	315186	0.33	1.04 (1.03, 1.06)	3.80E-06	410676	0.33	0.99 (0.97, 1.01)	0.24	410456
rs12906962	15	95312071	-	C	T	0.32	0.22 (0.16, 0.27)	2.10E-14	315186	0.33	1 (0.98, 1.02)	0.75	395066	0.33	1.01 (0.99, 1.03)	0.24	394846
rs4984497	15	96635899	-	T	C	0.33	0.2 (0.14, 0.25)	2.30E-12	315186	0.33	0.99 (0.9, 1.07)	0.74	15692	0.33	1.03 (0.95, 1.11)	0.49	15692
rs77924615	16	20392332	PDILT	G	A	0.8	0.21 (0.15, 0.28)	2.50E-10	315186	0.8	0.99 (0.96, 1.01)	0.24	410797	0.8	0.99 (0.97, 1.01)	0.45	410577
rs28545584	16	69329590	SNTB2	G	A	0.74	0.17 (0.11, 0.23)	1.60E-08	315186	0.75	1 (0.98, 1.02)	0.96	410809	0.75	0.98 (0.96, 1)	0.068	410589
rs576185956	17	2167393	SMG6	A	G	0.66	0.17 (0.11, 0.22)	1.90E-09	315186	0.66	1 (0.98, 1.02)	0.82	395066	0.66	1.01 (1, 1.03)	0.14	394846
rs9912501	17	3881345	ZZEF1	T	C	0.53	0.16 (0.11, 0.21)	6.80E-10	315186	0.53	1.01 (0.99, 1.02)	0.39	410763	0.53	1.01 (0.99, 1.02)	0.53	410543
rs4471743	17	4633653	MED11	G	A	0.22	0.18 (0.12, 0.24)	2.20E-08	315186	0.21	1 (0.97, 1.02)	0.75	395066	0.21	0.99 (0.97, 1.01)	0.32	394846
rs72824466	17	15381821	CDRT4; TVP23C-CDRT4; TVP23C	T	C	0.92	0.27 (0.18, 0.37)	4.60E-08	315186	0.93	0.99 (0.96, 1.03)	0.74	410712	0.93	1.03 (0.99, 1.06)	0.14	410492
rs2301597	17	43173273	NMT1	T	C	0.42	0.17 (0.12, 0.22)	1.60E-10	315186	0.44	1.03 (1.01, 1.05)	0.0032	395066	0.43	1 (0.98, 1.02)	0.76	394846
rs28394864	17	47450775	RP11-81K2.1	A	G	0.46	0.16 (0.11, 0.21)	3.10E-09	315186	0.46	1.02 (1, 1.04)	0.042	395066	0.46	1.02 (1.01, 1.04)	0.011	394846
rs147295858	17	61298602	TANC2	G	A	0.99	0.8 (0.53, 1.07)	5.90E-09	315186	0.99	0.95 (0.87, 1.04)	0.24	410732	0.99	1.01 (0.93, 1.1)	0.82	410512
rs12454132	18	10873221	PIEZO2	A	G	0.67	0.17 (0.11, 0.22)	1.70E-09	315186	0.69	0.99 (0.97, 1.01)	0.36	395066	0.69	0.99 (0.97, 1.01)	0.4	394846
rs61148001	18	48133241	MAPK4	C	T	0.79	0.19 (0.12, 0.25)	7.40E-09	315186	0.79	1.03 (1.01, 1.05)	0.017	395066	0.79	1 (0.97, 1.02)	0.73	394846
rs62107917	19	41136876	LTBP4	A	G	0.17	0.2 (0.13, 0.26)	3.70E-08	315186	0.17	1 (0.98, 1.02)	0.98	395066	0.17	1 (0.97, 1.02)	0.83	394846
rs73036520	19	45749484	MARK4	C	G	0.25	0.19 (0.13, 0.25)	9.10E-10	315186	0.25	1.03 (1.01, 1.05)	0.0036	395066	0.25	1.01 (0.99, 1.04)	0.19	394846
rs77289079	20	10743565	JAG1	G	T	0.73	0.3 (0.24, 0.36)	6.40E-24	315186	0.73	1.01 (0.99, 1.03)	0.56	395066	0.73	0.99 (0.97, 1.01)	0.42	394846
rs78473917	20	47420709	PREX1	T	C	0.85	0.23 (0.16, 0.3)	4.20E-10	315186	0.86	1.02 (1, 1.05)	0.11	410805	0.86	1 (0.98, 1.03)	0.94	410585
rs75777337	20	57702450	ZNF831	A	T	0.1	0.31 (0.22, 0.39)	7.40E-13	315186	0.11	1.02 (0.99, 1.05)	0.22	410695	0.11	1.01 (0.99, 1.04)	0.34	410475
rs9305545	21	35595821	-	G	A	0.16	0.21 (0.14, 0.28)	1.20E-08	315186	0.15	1.03 (1.01, 1.06)	0.012	410813	0.15	1.01 (0.99, 1.04)	0.32	410593
rs2836411	21	39819830	ERG	T	C	0.35	0.21 (0.15, 0.26)	1.20E-13	315186	0.35	0.99 (0.91, 1.08)	0.85	15638	0.35	1 (0.93, 1.08)	0.97	15638
rs134041	22	28056338	-	T	C	0.44	0.16 (0.1, 0.21)	5.70E-09	315186	0.44	1.01 (0.99, 1.02)	0.53	410738	0.44	0.98 (0.96, 1)	0.021	410518

Chr: Chromosome; EA: Effect Allele; NEA: Non-effect Allele; EAF: Effect Allele Frequency.
SNPs excluded in the MR-PRESSO analysis of HFrEF: rs11636952, rs72854462, rs9352694.
SNPs excluded in the MR-PRESSO analysis of HFpEF: none.

Table S4. GWAS Summary Used for Mendelian Randomization Analysis of Pulse Pressure (PP).

rsID	Chr	Position (hg19)	Gene	EA	NEA	SBP				HFrEF				HFpEF			
						EAF	Beta (95% CI)	P	N	EAF	OR (95% CI)	P	N	EAF	OR (95% CI)	P	N
rs9662255	1	9441949	SPSB1	C	A	0.58	0.26 (0.19, 0.33)	1.60E-13	315180	0.6	1 (0.99, 1.02)	0.69	410709	0.6	1.01 (1, 1.03)	0.12	410489
rs7516462	1	15972597	DDI2	C	T	0.74	0.23 (0.16, 0.31)	3.50E-09	315180	0.73	1.01 (0.99, 1.03)	0.23	395066	0.73	1.01 (0.99, 1.03)	0.21	394846
rs143167197	1	28734372	PHACTR4	G	A	0.07	0.38 (0.25, 0.52)	1.30E-08	315180	0.07	1.03 (1, 1.07)	0.075	395066	0.07	1 (0.96, 1.03)	0.79	394846
rs12403298	1	42359581	HIVEP3	A	T	0.09	0.38 (0.26, 0.5)	4.40E-10	315180	0.09	0.98 (0.95, 1.01)	0.19	410782	0.09	1.01 (0.98, 1.04)	0.47	410562
rs7546149	1	67101105	SGIP1	G	A	0.1	0.36 (0.25, 0.47)	2.70E-10	315180	0.1	1.03 (1, 1.07)	0.042	395066	0.1	1.02 (0.99, 1.05)	0.23	394846
rs12138150	1	169098738	ATP1B1	C	T	0.6	0.26 (0.19, 0.33)	2.40E-13	315180	0.62	1.01 (0.99, 1.03)	0.38	410759	0.62	1 (0.98, 1.02)	0.96	410539
rs12118102	1	176634724	PAPPA2	A	G	0.95	0.48 (0.33, 0.63)	3.20E-10	315180	0.95	0.98 (0.94, 1.02)	0.34	410814	0.95	1 (0.96, 1.03)	0.8	410594
rs536070	1	201748800	NAV1	C	T	0.4	0.21 (0.14, 0.28)	2.70E-09	315180	0.38	1 (0.98, 1.02)	0.81	395066	0.38	1 (0.98, 1.02)	0.77	394846
rs10494852	1	204457786	PIK3C2B	C	T	0.31	0.21 (0.13, 0.28)	2.40E-08	315180	0.32	0.99 (0.97, 1.01)	0.16	410698	0.32	0.98 (0.96, 1)	0.026	410478
rs12063025	1	208123610	CD34	C	A	0.39	0.25 (0.18, 0.32)	7.20E-13	315180	0.38	0.99 (0.97, 1.01)	0.37	395066	0.38	1.01 (0.99, 1.03)	0.35	394846
rs12133169	1	219792380	-	G	A	0.76	0.29 (0.21, 0.37)	7.90E-13	315180	0.77	1.01 (0.98, 1.03)	0.64	395066	0.77	1 (0.98, 1.02)	0.97	394846
rs7255	2	20878820	C2orf43	C	T	0.56	0.27 (0.2, 0.34)	5.50E-15	315180	0.53	1 (0.99, 1.02)	0.62	410734	0.53	1.01 (0.99, 1.02)	0.53	410514
rs1275984	2	26911509	KCNK3	A	C	0.38	0.21 (0.14, 0.28)	2.90E-09	315180	0.39	1.01 (0.99, 1.03)	0.35	395066	0.39	1.02 (1, 1.03)	0.097	394846
rs848558	2	36694559	CRIM1	T	C	0.27	0.22 (0.15, 0.3)	1.30E-08	315180	0.26	0.99 (0.97, 1.01)	0.2	410701	0.26	0.99 (0.97, 1.01)	0.27	410481
rs11690961	2	46363336	PRKCE	A	C	0.88	0.39 (0.29, 0.49)	2.60E-13	315180	0.89	0.98 (0.96, 1.01)	0.19	410709	0.89	1.02 (0.99, 1.05)	0.19	410489
rs10865317	2	60557031	-	G	A	0.4	0.2 (0.13, 0.27)	2.00E-08	315180	0.38	0.99 (0.97, 1.01)	0.45	395066	0.38	0.99 (0.97, 1.01)	0.33	394846
rs72816660	2	79407110	CTNNA2	T	C	0.14	0.29 (0.2, 0.39)	2.60E-09	315180	0.12	1.03 (1.01, 1.07)	0.021	395066	0.12	1.01 (0.98, 1.04)	0.56	394846
rs11126989	2	85490112	TCF7L1	T	A	0.54	0.27 (0.21, 0.34)	2.10E-15	315180	0.55	1.02 (1.01, 1.04)	0.011	410749	0.55	1 (0.98, 1.01)	0.65	410529
rs2969489	2	96811434	DUSP2	T	C	0.43	0.27 (0.2, 0.34)	7.20E-15	315180	0.45	1 (0.98, 1.01)	0.75	410763	0.45	1 (0.98, 1.01)	0.75	410543
rs13403907	2	145222038	ZEB2	G	A	0.13	0.3 (0.19, 0.4)	1.40E-08	315180	0.12	1.04 (1.01, 1.06)	0.012	395066	0.12	1.04 (1.01, 1.07)	0.0039	394846
rs12620124	2	164940271	-	C	T	0.83	0.47 (0.38, 0.56)	2.80E-25	315180	0.83	0.98 (0.96, 1.01)	0.13	410679	0.83	1 (0.97, 1.02)	0.79	410459
rs560887	2	169763148	SPC25; G6PC2	C	T	0.7	0.32 (0.25, 0.39)	8.70E-18	315180	0.7	1.01 (0.99, 1.03)	0.22	410809	0.7	0.99 (0.97, 1.01)	0.46	410589
rs115150884	2	179793839	CCDC141	C	G	0.96	0.64 (0.46, 0.83)	6.70E-12	315180	0.96	1.02 (0.97, 1.07)	0.46	395066	0.96	0.9 (0.86, 0.95)	1.80E-05	394846
rs1250259	2	216300482	FN1	T	A	0.26	0.36 (0.28, 0.43)	6.50E-20	315180	0.26	1.06 (0.97, 1.16)	0.21	15707	0.26	0.94 (0.87, 1.02)	0.16	15707
rs2643826	3	27562988	SLC4A7	T	C	0.45	0.22 (0.15, 0.29)	2.20E-10	315180	0.45	1.03 (1.01, 1.05)	0.0019	395066	0.45	1.01 (1, 1.03)	0.13	394846
rs6801957	3	38767315	SCN10A	T	C	0.41	0.22 (0.15, 0.29)	3.30E-10	315180	0.41	0.98 (0.96, 1)	0.019	410809	0.41	0.98 (0.96, 1)	0.032	410589
rs7649989	3	41865843	ULK4	A	C	0.84	0.71 (0.62, 0.8)	7.70E-52	315180	0.83	0.98 (0.96, 1)	0.11	410813	0.83	1 (0.98, 1.02)	0.91	410593
rs2241823	3	63965093	ATXN7	C	A	0.7	0.24 (0.17, 0.32)	1.10E-10	315180	0.69	1.01 (0.99, 1.03)	0.29	395066	0.69	1 (0.98, 1.02)	0.9	394846
rs9840088	3	66442545	LRIG1	A	C	0.8	0.27 (0.18, 0.35)	4.60E-10	315180	0.8	0.98 (0.95, 1)	0.033	410798	0.8	1 (0.98, 1.02)	0.85	410578
rs1599116	3	115077351	-	G	T	0.14	0.28 (0.18, 0.38)	1.20E-08	315180	0.16	1.02 (0.99, 1.04)	0.15	410735	0.16	1.02 (1, 1.05)	0.068	410515

rs55914222	3	128202943	GATA2	C	G	0.03	0.64 (0.44, 0.85)	5.00E-10	315180	0.03	1.09 (1.02, 1.16)	0.0081	395066	0.03	1.03 (0.96, 1.1)	0.39	394846
rs1470121	3	142625778	PCOLCE2	G	A	0.64	0.2 (0.13, 0.27)	1.60E-08	315180	0.65	1.01 (0.99, 1.03)	0.44	410664	0.65	1.01 (0.99, 1.03)	0.46	410444
rs231708	4	2694773	FAM193A	G	C	0.31	0.2 (0.13, 0.28)	4.00E-08	315180	0.31	1.03 (1.01, 1.05)	0.0035	395066	0.31	1.03 (1.01, 1.05)	0.0077	394846
rs2498323	4	3451109	HGFAC	A	G	0.1	0.36 (0.24, 0.47)	4.60E-10	315180	0.1	1.03 (1, 1.06)	0.072	410763	0.1	1 (0.97, 1.03)	0.85	410543
rs6838613	4	48476539	SLC10A4	A	G	0.48	0.23 (0.16, 0.3)	1.60E-11	315180	0.48	1.01 (0.99, 1.03)	0.25	395066	0.48	0.99 (0.98, 1.01)	0.44	394846
rs12502173	4	56534881	NMU	A	C	0.1	0.33 (0.21, 0.44)	1.70E-08	315180	0.08	1 (0.97, 1.03)	0.94	395066	0.08	1.03 (0.99, 1.06)	0.13	394846
rs79980676	4	86746256	ARHGAP24	A	G	0.11	0.41 (0.3, 0.52)	6.10E-14	315180	0.1	1 (0.98, 1.03)	0.77	395066	0.1	0.99 (0.96, 1.02)	0.62	394846
rs11726786	4	106120756	TET2	G	T	0.37	0.2 (0.13, 0.27)	2.50E-08	315180	0.35	0.99 (0.97, 1.01)	0.18	410746	0.35	0.98 (0.96, 1)	0.04	410526
rs1979974	4	146800815	ZNF827	A	G	0.52	0.21 (0.15, 0.28)	4.20E-10	315180	0.53	0.97 (0.9, 1.06)	0.55	15729	0.53	1.06 (0.98, 1.13)	0.14	15729
rs17612742	4	148414651	EDNRA	C	T	0.14	0.3 (0.2, 0.4)	2.50E-09	315180	0.13	1.04 (1.02, 1.07)	0.0012	410792	0.13	1.01 (0.99, 1.04)	0.36	410572
rs17033050	4	156401693	-	G	A	0.16	0.37 (0.28, 0.46)	2.60E-15	315180	0.15	1.01 (0.99, 1.04)	0.23	410813	0.15	1.01 (0.98, 1.03)	0.62	410593
rs7696431	4	169687725	PALLD	T	G	0.52	0.3 (0.23, 0.36)	8.50E-18	315180	0.52	1.02 (1.01, 1.04)	0.009	395066	0.52	1 (0.98, 1.01)	0.68	394846
rs62360778	5	108201844	FER	T	G	0.79	0.26 (0.17, 0.34)	1.00E-09	315180	0.79	1.01 (0.99, 1.03)	0.54	410713	0.79	1.01 (0.99, 1.03)	0.24	410493
rs258800	5	142532696	ARHGAP26	G	A	0.41	0.21 (0.14, 0.27)	4.30E-09	315180	0.4	0.99 (0.97, 1.01)	0.21	395066	0.4	0.99 (0.97, 1.01)	0.28	394846
rs4705300	5	148336213	SH3TC2	A	G	0.33	0.21 (0.13, 0.28)	1.90E-08	315180	0.33	1.01 (0.99, 1.03)	0.23	395066	0.33	1 (0.98, 1.02)	0.94	394846
rs157678	5	156145654	SGCD	T	A	0.34	0.21 (0.14, 0.29)	5.30E-09	315180	0.33	0.99 (0.97, 1.01)	0.16	395066	0.33	1 (0.98, 1.02)	0.89	394846
rs742556	6	1633682	GMDS	C	T	0.3	0.2 (0.13, 0.28)	4.80E-08	315180	0.3	0.98 (0.96, 0.99)	0.011	410769	0.3	1 (0.98, 1.02)	0.91	410549
rs6920534	6	7519183	DSP	T	C	0.9	0.35 (0.23, 0.46)	2.00E-09	315180	0.91	0.99 (0.95, 1.02)	0.35	410802	0.91	1 (0.97, 1.04)	0.82	410582
rs9349379	6	12903957	PHACTR1	A	G	0.6	0.32 (0.25, 0.39)	2.50E-20	315180	0.6	0.94 (0.92, 0.95)	6.70E-13	410785	0.6	1 (0.98, 1.02)	0.96	410565
rs236349	6	36820565	PPIL1	A	G	0.34	0.21 (0.14, 0.28)	7.70E-09	315180	0.35	1.01 (0.99, 1.03)	0.34	395066	0.35	1 (0.98, 1.02)	0.75	394846
rs9363415	6	62597252	KHDRBS2	C	T	0.51	0.21 (0.15, 0.28)	4.90E-10	315180	0.5	1 (0.98, 1.01)	0.65	395066	0.5	1.02 (1, 1.03)	0.08	394846
rs4513767	6	63675650	-	C	T	0.38	0.19 (0.12, 0.26)	4.90E-08	315180	0.39	0.99 (0.98, 1.01)	0.5	410695	0.39	0.99 (0.97, 1.01)	0.24	410475
rs16875178	6	85516100	TBX18	T	C	0.91	0.37 (0.25, 0.49)	2.30E-09	315180	0.92	1.01 (0.98, 1.04)	0.54	395066	0.92	1.03 (1, 1.07)	0.073	394846
rs9486719	6	97060124	FHL5	A	G	0.2	0.27 (0.19, 0.36)	2.80E-10	315180	0.21	1.02 (0.92, 1.13)	0.64	15687	0.21	0.99 (0.91, 1.08)	0.84	15687
rs11752784	6	122193876	GJA1	A	G	0.9	0.54 (0.43, 0.66)	2.10E-20	315180	0.9	0.99 (0.96, 1.02)	0.5	395066	0.9	1.02 (0.98, 1.05)	0.33	394846
rs6941056	6	143591821	AIG1	C	G	0.56	0.26 (0.2, 0.33)	2.20E-14	315180	0.56	1 (0.99, 1.02)	0.63	395066	0.56	0.99 (0.97, 1.01)	0.42	394846
rs13219476	6	152325504	ESR1	G	T	0.1	0.64 (0.53, 0.75)	5.00E-30	315180	0.1	1.03 (1, 1.06)	0.098	410724	0.1	1.03 (1, 1.06)	0.073	410504
rs11754038	6	155567880	TIAM2	T	C	0.62	0.22 (0.15, 0.29)	8.40E-10	315180	0.6	1.01 (0.99, 1.02)	0.53	410674	0.6	0.97 (0.96, 0.99)	0.0025	410454
rs434578	6	159693220	FNDC1	C	T	0.14	0.46 (0.36, 0.55)	2.20E-20	315180	0.15	1.01 (0.98, 1.04)	0.46	395066	0.15	1.03 (1, 1.06)	0.052	394846
rs1322639	6	169587103	THBS2	A	G	0.78	0.4 (0.32, 0.48)	4.90E-22	315180	0.76	1.01 (0.99, 1.04)	0.2	410707	0.76	1 (0.98, 1.02)	0.85	410487
rs2107595	7	19049388	HDAC9	A	G	0.15	0.53 (0.43, 0.62)	5.20E-28	315180	0.17	1.02 (1, 1.05)	0.045	410639	0.17	1.02 (0.99, 1.04)	0.15	410419
rs6461992	7	27220831	HOXA11	G	A	0.93	0.41 (0.28, 0.54)	6.50E-10	315180	0.91	1.05 (1.02, 1.09)	0.0048	395066	0.91	1.04 (1.01, 1.08)	0.018	394846
rs4723954	7	40417925	SUGCT	G	A	0.89	0.35 (0.24, 0.46)	3.50E-10	315180	0.88	0.98 (0.95, 1.01)	0.11	410763	0.88	1.01 (0.98, 1.03)	0.67	410543
rs10248254	7	88550492	ZNF804B	G	A	0.93	0.39 (0.26, 0.52)	5.90E-09	315180	0.92	0.99 (0.96, 1.02)	0.6	410798	0.92	1 (0.97, 1.04)	0.87	410578

rs12539008	7	89832200	STEAP2	C	T	0.53	0.23 (0.16, 0.3)	2.80E-11	315180	0.52	1 (0.98, 1.02)	0.83	395066	0.52	1 (0.98, 1.02)	0.93	394846
rs2282978	7	92264410	CDK6	T	C	0.68	0.47 (0.39, 0.54)	9.20E-37	315180	0.66	1.04 (1.02, 1.05)	0.00015	410814	0.66	1.02 (1, 1.04)	0.015	410594
rs3807989	7	116186241	CAV1	A	G	0.41	0.22 (0.15, 0.29)	3.30E-10	315180	0.41	0.96 (0.95, 0.98)	1.40E-05	410811	0.41	0.99 (0.97, 1.01)	0.26	410591
rs2129561	7	130963771	MKLN1	G	A	0.6	0.21 (0.14, 0.28)	2.70E-09	315180	0.57	1 (0.98, 1.02)	0.81	410777	0.57	0.99 (0.97, 1.01)	0.42	410557
rs73727605	7	149474622	ZNF467	A	G	0.07	0.4 (0.27, 0.54)	3.90E-09	315180	0.06	1.01 (0.97, 1.05)	0.53	395066	0.06	1.01 (0.97, 1.05)	0.64	394846
rs73205721	8	13224375	DLC1	G	C	0.82	0.27 (0.18, 0.36)	1.50E-09	315180	0.82	1.02 (1, 1.05)	0.032	410716	0.82	1 (0.98, 1.03)	0.72	410496
rs1449544	8	76591880	-	A	C	0.55	0.24 (0.17, 0.31)	2.70E-12	315180	0.54	1 (0.98, 1.02)	0.93	395066	0.54	1.02 (1, 1.04)	0.035	394846
rs11991823	8	92188440	LRRC69	G	A	0.68	0.2 (0.13, 0.28)	3.40E-08	315180	0.67	1 (0.98, 1.02)	0.74	410736	0.67	1.01 (0.99, 1.03)	0.28	410516
rs1474516	8	116955558	-	A	G	0.17	0.27 (0.18, 0.36)	4.60E-09	315180	0.17	0.99 (0.97, 1.01)	0.43	410755	0.17	0.98 (0.96, 1)	0.1	410535
rs4288356	8	141055724	TRAPPC9	G	A	0.37	0.3 (0.23, 0.37)	1.60E-17	315180	0.38	0.99 (0.98, 1.01)	0.48	395066	0.38	1.01 (0.99, 1.03)	0.18	394846
rs1333045	9	22119195	-	C	T	0.51	0.23 (0.17, 0.3)	1.10E-11	315180	-	-	-	-	0.51	1.04 (1.02, 1.06)	8.90E-06	410571
rs989393	9	101743336	COL15A1	T	C	0.71	0.23 (0.16, 0.31)	9.80E-10	315180	0.71	1 (0.98, 1.02)	0.99	410799	0.71	1 (0.98, 1.02)	0.79	410579
rs60692488	9	123643427	PHF19	G	T	0.33	0.2 (0.13, 0.27)	4.20E-08	315180	0.33	1.01 (0.99, 1.03)	0.41	395066	0.33	1 (0.98, 1.02)	0.82	394846
rs72765277	9	127858106	SCAI	C	A	0.13	0.38 (0.28, 0.47)	7.50E-14	315180	0.13	1.01 (0.98, 1.03)	0.57	410780	0.13	1.04 (1.01, 1.06)	0.0052	410560
rs12780155	10	12261852	CDC123	A	T	0.2	0.26 (0.18, 0.34)	1.40E-09	315180	0.19	1.02 (1, 1.04)	0.11	410705	0.19	1 (0.98, 1.03)	0.81	410485
rs4748701	10	21012806	NEBL	G	A	0.35	0.21 (0.14, 0.28)	1.10E-08	315180	0.35	1 (0.98, 1.02)	0.95	410789	0.35	1 (0.98, 1.02)	0.83	410569
rs12221321	10	22802349	PIP4K2A	A	G	0.76	0.22 (0.14, 0.3)	3.60E-08	315180	0.77	1.01 (0.99, 1.03)	0.24	395066	0.77	1.01 (0.99, 1.03)	0.42	394846
rs11813059	10	31369709	ZNF438	T	G	0.71	0.26 (0.18, 0.33)	1.90E-11	315180	0.71	1.02 (1, 1.04)	0.02	410718	0.71	1.01 (0.99, 1.02)	0.6	410498
rs72786124	10	33377979	ITGB1	T	A	0.01	0.81 (0.52, 1.09)	4.70E-08	315180	0.01	0.99 (0.9, 1.08)	0.75	395066	0.01	0.95 (0.87, 1.04)	0.26	394846
rs11006192	10	60343638	BICC1	C	T	0.55	0.23 (0.16, 0.3)	2.30E-11	315180	0.53	1.01 (0.99, 1.03)	0.29	395066	0.53	1.02 (1, 1.04)	0.056	394846
rs7095472	10	70399109	TET1	A	G	0.47	0.2 (0.13, 0.27)	6.40E-09	315180	0.48	1.02 (1, 1.04)	0.03	395066	0.48	1.01 (1, 1.03)	0.13	394846
rs55947600	10	75797672	VCL	G	A	0.54	0.27 (0.2, 0.33)	9.20E-15	315180	0.54	1.01 (0.99, 1.03)	0.41	395066	0.54	1.01 (0.99, 1.02)	0.52	394846
rs11187844	10	96056629	PLCE1	C	A	0.89	0.38 (0.27, 0.49)	3.00E-12	315180	0.88	1.02 (1, 1.05)	0.078	395066	0.88	0.99 (0.97, 1.02)	0.6	394846
rs11191156	10	103702763	C10orf76	G	A	0.35	0.21 (0.14, 0.28)	7.50E-09	315180	0.35	1.01 (0.99, 1.03)	0.17	410812	0.35	1.01 (0.99, 1.03)	0.23	410592
rs6585201	10	114768783	TCF7L2	A	G	0.45	0.22 (0.15, 0.28)	4.10E-10	315180	0.45	1.01 (0.99, 1.03)	0.34	395066	0.45	0.99 (0.97, 1.01)	0.31	394846
rs686722	11	1891722	LSP1	T	C	0.35	0.29 (0.22, 0.37)	2.20E-16	315180	0.36	1.04 (1.02, 1.06)	9.80E-06	395066	0.36	1.01 (0.99, 1.03)	0.29	394846
rs10734252	11	17404839	KCNJ11	G	A	0.38	0.23 (0.17, 0.3)	3.40E-11	315180	0.39	1.02 (1, 1.04)	0.014	410749	0.39	1.01 (0.99, 1.02)	0.55	410529
rs4922591	11	32374199	-	C	T	0.62	0.2 (0.13, 0.27)	3.70E-08	315180	0.6	1.02 (1, 1.04)	0.02	395066	0.6	1 (0.98, 1.02)	0.73	394846
rs3740781	11	45255926	SYT13	T	C	0.74	0.31 (0.24, 0.39)	2.00E-15	315180	0.72	1 (0.98, 1.02)	0.9	395066	0.72	1.04 (1.02, 1.06)	0.00018	394846
rs7107356	11	47676170	AGBL2	G	A	0.51	0.2 (0.13, 0.26)	8.70E-09	315180	0.5	1.01 (0.99, 1.03)	0.37	410751	0.5	0.98 (0.97, 1)	0.036	410531
rs72931748	11	69825414	-	A	G	0.91	0.34 (0.22, 0.46)	1.40E-08	315180	0.92	0.99 (0.96, 1.03)	0.75	410682	0.92	1 (0.97, 1.03)	0.94	410462
rs7124280	11	102018263	YAP1	G	A	0.65	0.21 (0.14, 0.28)	4.50E-09	315180	0.66	1.01 (0.99, 1.03)	0.27	410799	0.66	1.01 (0.99, 1.03)	0.24	410579
rs4754196	11	107096777	CWF19L2	G	A	0.48	0.24 (0.17, 0.31)	1.90E-12	315180	0.47	1.03 (1.01, 1.04)	0.0036	410768	0.47	1 (0.98, 1.02)	0.96	410548
rs573455	11	117267884	CEP164	A	G	0.47	0.31 (0.24, 0.38)	2.40E-19	315180	0.46	0.99 (0.97, 1)	0.13	395066	0.46	1.01 (0.99, 1.03)	0.25	394846

rs78799967	11	118266523	UBE4A	C	T	0.97	0.65 (0.44, 0.87)	2.30E-09	315180	0.98	1.04 (0.96, 1.13)	0.35	395066	0.98	0.96 (0.89, 1.05)	0.37	394846
rs4937515	11	130268147	ADAMT S8	G	C	0.41	0.6 (0.53, 0.67)	1.10E-65	315180	0.4	0.99 (0.97, 1.01)	0.26	395066	0.4	1 (0.98, 1.02)	0.91	394846
rs11054925	12	12625848	DUSP16	T	C	0.66	0.21 (0.14, 0.28)	6.30E-09	315180	0.66	1.02 (1, 1.04)	0.067	395066	0.66	1.02 (1, 1.04)	0.1	394846
rs10770612	12	20230639	-	A	G	0.8	0.39 (0.31, 0.48)	4.40E-20	315180	0.8	0.99 (0.97, 1.01)	0.23	410789	0.8	1 (0.98, 1.03)	0.7	410569
rs11047543	12	24788339	-	A	G	0.15	0.28 (0.19, 0.38)	2.90E-09	315180	0.15	1 (0.97, 1.02)	0.86	410810	0.15	1 (0.97, 1.02)	0.8	410590
rs7296714	12	27397008	STK38L	G	C	0.17	0.26 (0.17, 0.34)	2.30E-08	315180	0.17	1.02 (0.99, 1.04)	0.18	410812	0.17	1.01 (0.98, 1.03)	0.53	410592
rs4587807	12	33510115	SYT10	A	G	0.5	0.22 (0.16, 0.29)	9.40E-11	315180	0.51	1.01 (0.99, 1.02)	0.44	410805	0.51	1 (0.99, 1.02)	0.79	410585
rs11105352	12	90026462	ATP2B1	G	A	0.83	0.4 (0.31, 0.49)	8.50E-18	315180	0.83	0.96 (0.94, 0.98)	0.00019	410809	0.83	1.02 (0.99, 1.04)	0.17	410589
rs10859566	12	94126098	CRADD	A	G	0.45	0.22 (0.16, 0.29)	1.20E-10	315180	0.43	0.99 (0.97, 1.01)	0.35	410754	0.43	1.01 (0.99, 1.02)	0.55	410534
rs114697502	12	94677559	PLXNC1	C	T	0.91	0.41 (0.29, 0.53)	1.60E-11	315180	0.93	1.04 (1, 1.07)	0.029	395066	0.93	1.04 (1, 1.07)	0.039	394846
rs7954260	12	95518223	FGD6	T	C	0.82	0.28 (0.2, 0.37)	1.60E-10	315180	0.81	1.03 (1.01, 1.05)	0.012	410673	0.81	1.02 (1, 1.04)	0.082	410453
rs686090	13	110795080	COL4A1	T	A	0.09	0.35 (0.23, 0.47)	3.40E-09	315180	0.1	0.98 (0.95, 1.01)	0.25	395066	0.1	1.01 (0.98, 1.04)	0.63	394846
rs9549328	13	113636156	MCF2L	T	C	0.23	0.25 (0.17, 0.33)	2.10E-09	315180	0.23	1.03 (1, 1.05)	0.021	395066	0.23	0.99 (0.97, 1.02)	0.57	394846
rs11623972	14	73381520	DCAF4	A	G	0.36	0.27 (0.2, 0.34)	6.60E-14	315180	0.39	1 (0.98, 1.02)	0.74	395066	0.39	1 (0.98, 1.02)	0.74	394846
rs34237661	14	94465413	-	G	A	0.68	0.22 (0.15, 0.3)	2.00E-09	315180	0.71	1 (0.98, 1.02)	0.77	410774	0.71	0.99 (0.98, 1.01)	0.57	410554
rs1121703	14	98552503	-	T	G	0.4	0.24 (0.17, 0.31)	4.30E-12	315180	0.41	1.01 (0.99, 1.03)	0.29	395066	0.41	1.03 (1.01, 1.05)	0.00061	394846
rs17562391	14	100133250	HHIPL1	T	C	0.42	0.23 (0.17, 0.3)	1.50E-11	315180	0.41	1.01 (0.99, 1.03)	0.21	410809	0.41	1.01 (0.99, 1.03)	0.29	410589
rs11627794	14	104140915	KLC1; RP11-73M18.2	T	C	0.25	0.22 (0.15, 0.3)	2.30E-08	315180	0.24	0.98 (0.96, 1)	0.019	395066	0.24	0.98 (0.96, 1)	0.099	394846
rs1036477	15	48914926	FBN1	A	G	0.9	0.57 (0.46, 0.68)	2.90E-23	315180	0.88	0.99 (0.96, 1.02)	0.56	410813	0.88	0.98 (0.95, 1.01)	0.12	410593
rs4337253	15	71609306	THSD4	C	G	0.33	0.22 (0.15, 0.29)	1.60E-09	315180	0.33	1.01 (0.99, 1.03)	0.27	395066	0.33	1.02 (1, 1.04)	0.069	394846
rs2071382	15	91428197	FES	T	C	0.47	0.24 (0.17, 0.31)	3.10E-12	315180	0.47	1.02 (1, 1.04)	0.017	395066	0.47	0.99 (0.98, 1.01)	0.51	394846
rs893373	15	93246339	FAM174 B	G	A	0.37	0.2 (0.13, 0.27)	1.60E-08	315180	0.38	0.98 (0.96, 1)	0.04	395066	0.38	0.98 (0.96, 1)	0.069	394846
rs374179645	16	65272050	-	G	A	0.57	0.29 (0.22, 0.36)	4.40E-17	315180	0.56	1 (0.98, 1.02)	0.9	395066	0.56	0.98 (0.97, 1)	0.077	394846
rs11650511	17	1337960	CRK	T	C	0.44	0.22 (0.16, 0.29)	1.00E-10	315180	0.43	1.02 (1, 1.04)	0.067	395066	0.43	1.01 (0.99, 1.03)	0.45	394846
rs9912392	17	1995611	SMG6	G	C	0.42	0.24 (0.17, 0.31)	3.10E-12	315180	0.43	0.97 (0.96, 0.99)	0.0059	395066	0.43	0.99 (0.98, 1.01)	0.48	394846
rs1990288	17	26815328	RP11-192H23.4 ; SLC13A2	G	A	0.78	0.23 (0.15, 0.31)	4.90E-08	315180	0.79	0.99 (0.97, 1.01)	0.43	395066	0.79	0.99 (0.96, 1.01)	0.22	394846
rs35447453	17	40132591	DNAJC7	A	G	0.12	0.36 (0.26, 0.47)	7.60E-12	315180	0.1	1.03 (0.99, 1.06)	0.11	395066	0.1	1 (0.97, 1.03)	0.98	394846
rs71373532	17	43153235	NMT1	T	C	0.08	0.52 (0.39, 0.65)	1.40E-15	315180	0.07	1.05 (1.01, 1.08)	0.015	395066	0.07	1 (0.97, 1.04)	0.89	394846
rs17608766	17	45013271	GOSR2; RP11-156P1.2	C	T	0.15	0.5 (0.41, 0.6)	2.00E-25	315180	0.13	1.03 (1, 1.06)	0.023	395066	0.13	1.03 (1.01, 1.06)	0.019	394846
rs1292052	17	57963873	TUBD1	C	T	0.89	0.31 (0.2, 0.42)	3.00E-08	315180	0.88	1.01 (0.98, 1.04)	0.66	395066	0.88	1.03 (1, 1.06)	0.056	394846
rs3786130	17	60765043	MRC2	C	T	0.43	0.29 (0.22, 0.36)	7.00E-17	315180	0.41	1.01 (0.99, 1.03)	0.36	410794	0.41	1.01 (1, 1.03)	0.12	410574
rs8090594	18	42514448	SETBP1	G	A	0.47	0.19 (0.12, 0.26)	3.70E-08	315180	0.46	1 (0.98, 1.02)	0.9	410811	0.46	1.01 (0.99, 1.02)	0.52	410591

rs7236548	18	43097750	SLC14A2	A	C	0.18	0.44 (0.35, 0.53)	8.50E-23	315180	0.18	1.01 (0.99, 1.03)	0.37	410693	0.18	1.01 (0.99, 1.04)	0.3	410473
rs8102624	19	2161443	AP3D1	A	G	0.07	0.63 (0.5, 0.76)	2.70E-21	315180	0.08	0.96 (0.93, 0.99)	0.015	395066	0.08	1.02 (0.99, 1.06)	0.16	394846
rs1982468	19	4953351	KDM4B	T	G	0.64	0.23 (0.15, 0.3)	3.80E-10	315180	0.64	1.01 (0.99, 1.03)	0.35	410759	0.64	1.01 (0.99, 1.03)	0.37	410539
rs12982634	19	7233193	INSR	C	T	0.68	0.26 (0.19, 0.33)	1.60E-12	315180	0.69	1 (0.98, 1.02)	0.67	395066	0.69	1 (0.98, 1.02)	0.87	394846
rs2116941	19	10334443	DNMT1	A	C	0.19	0.24 (0.16, 0.33)	3.40E-08	315180	0.21	1.01 (0.98, 1.03)	0.54	395066	0.21	1.01 (0.99, 1.03)	0.38	394846
rs7408868	19	15285679	NOTCH3	C	G	0.9	0.49 (0.38, 0.61)	1.50E-17	315180	0.88	1.02 (0.99, 1.05)	0.29	395066	0.88	1.02 (0.99, 1.06)	0.11	394846
rs1165840	19	41077501	SPTBN4	C	T	0.65	0.22 (0.15, 0.29)	1.60E-09	315180	0.64	1 (0.98, 1.02)	0.84	395066	0.64	1.01 (0.99, 1.03)	0.26	394846
rs1065853	19	45413233	APOE	G	T	0.92	0.34 (0.22, 0.47)	4.50E-08	315180	0.92	1.04 (1, 1.07)	0.028	410789	0.92	1.02 (0.98, 1.05)	0.31	410569
rs8103278	19	46370381	FOXA3	G	A	0.65	0.22 (0.15, 0.29)	1.40E-09	315180	0.65	0.99 (0.97, 1.01)	0.2	395066	0.65	0.98 (0.96, 1)	0.018	394846
rs742707	20	2777828	CPXM1	G	T	0.42	0.2 (0.13, 0.27)	1.00E-08	315180	0.44	1.02 (1, 1.04)	0.013	395066	0.44	0.99 (0.97, 1.01)	0.42	394846
rs6035355	20	19465089	SLC24A3	A	T	0.28	0.37 (0.29, 0.44)	2.50E-21	315180	0.28	1 (0.91, 1.1)	0.96	15735	0.28	1.04 (0.96, 1.13)	0.33	15735
rs6062533	20	62429101	ZBTB46	G	A	0.77	0.24 (0.16, 0.32)	3.50E-09	315180	0.76	1.03 (1.01, 1.05)	0.01	395066	0.76	1.03 (1.01, 1.05)	0.0072	394846
rs57448815	21	30123533	-	G	A	0.14	0.36 (0.27, 0.46)	2.10E-13	315180	0.14	0.99 (0.97, 1.02)	0.45	410814	0.14	1.03 (1, 1.05)	0.041	410594
rs117870289	21	39983448	ERG	T	C	0.02	1.25 (0.98, 1.52)	1.50E-19	315180	0.01	0.9 (0.82, 1)	0.046	395066	0.01	0.99 (0.89, 1.09)	0.78	394846
rs4819852	22	19988167	ARVCF	A	G	0.28	0.22 (0.14, 0.29)	1.10E-08	315180	0.28	1 (0.98, 1.02)	0.71	395066	0.28	1.01 (0.99, 1.03)	0.35	394846
rs9607706	22	40740104	ADSL	A	G	0.18	0.31 (0.22, 0.4)	7.40E-12	315180	0.18	0.99 (0.97, 1.01)	0.31	410779	0.18	1 (0.98, 1.02)	0.96	410559

Chr: Chromosome; EA: Effect Allele; NEA: Non-effect Allele; EAF: Effect Allele Frequency.

SNPs excluded in the MR-PRESSO analysis of HFrEF: rs11105352, rs3807989, rs686722, rs9349379.

SNPs excluded in the MR-PRESSO analysis of HFpEF: rs115150884, rs11754038, rs1333045, rs3740781.

Table S5. GWAS Summary Used for Mendelian Randomization Analysis of High-density Lipoprotein Cholesterol (HDL-C).

rsID	Chr	Position (hg19)	Gene	EA	NEA	HDL-C				HFrEF				HFpEF			
						EAF	Beta (95% CI)	P	N	EAF	OR (95% CI)	P	N	EAF	OR (95% CI)	P	N
rs2298214	1	935222	HES4	C	A	0.42	0.21 (0.14, 0.28)	1.60E-09	294484	0.44	0.99 (0.97, 1.01)	0.32	395066	0.44	0.99 (0.98, 1.01)	0.57	394846
rs193084249	1	26987646	ARID1A	A	G	0.98	1.1 (0.87, 1.33)	2.60E-21	294484	0.98	0.95 (0.89, 1.01)	0.12	395066	0.98	0.98 (0.92, 1.05)	0.54	394846
rs72663520	1	40003410	BMP8A	C	T	0.77	0.62 (0.54, 0.7)	2.30E-50	294484	0.78	0.99 (0.97, 1.01)	0.36	395066	0.78	0.96 (0.94, 0.98)	0.00011	394846
rs4344355	1	63153953	DOCK7	C	T	0.65	0.26 (0.19, 0.34)	6.60E-13	294484	0.67	1.02 (1, 1.04)	0.045	410764	0.67	1.01 (0.99, 1.03)	0.34	410544
rs6664374	1	66073952	LEPR	T	C	0.35	0.25 (0.17, 0.32)	1.70E-11	294484	0.35	1.01 (0.99, 1.03)	0.45	395066	0.35	1 (0.98, 1.01)	0.67	394846
rs35628716	1	93839608	DR1	T	G	0.18	0.43 (0.34, 0.51)	1.70E-21	294484	0.18	0.98 (0.96, 1)	0.071	410733	0.18	1.01 (0.98, 1.03)	0.6	410513
rs646776	1	109818530	CELSR2	C	T	0.22	0.32 (0.24, 0.41)	7.60E-15	294484	0.22	0.94 (0.92, 0.96)	9.90E-10	410812	0.22	0.97 (0.95, 0.99)	0.00074	410592
rs140584594	1	110232983	GSTM2; GSTM1	G	A	0.73	0.46 (0.38, 0.53)	8.50E-32	294484	0.04	1.05 (0.98, 1.12)	0.15	395066	0.04	0.94 (0.88, 1.01)	0.079	394846
rs115298177	1	150878649	SETDB1	T	C	0.04	0.68 (0.5, 0.86)	2.90E-13	294484	0.05	0.98 (0.94, 1.02)	0.38	395066	0.05	1.02 (0.97, 1.06)	0.43	394846
rs1771582	1	161614490	FCGR2B	G	T	0.56	0.24 (0.16, 0.31)	7.20E-11	294484	0.54	1.04 (1.02, 1.06)	2.40E-05	395066	0.54	1 (0.98, 1.02)	0.81	394846
rs10798615	1	178513895	TEX35; C1orf220; C1ORF220	T	G	0.47	0.29 (0.22, 0.36)	1.00E-16	294484	0.48	1 (0.98, 1.01)	0.72	410808	0.48	0.99 (0.98, 1.01)	0.45	410588
rs7522056	1	205735891	RAB7L1	G	A	0.66	0.2 (0.13, 0.28)	2.80E-08	294484	0.68	0.99 (0.98, 1.01)	0.51	410709	0.68	0.97 (0.96, 0.99)	0.0066	410489
rs12034581	1	219694903	-	C	A	0.49	0.21 (0.14, 0.28)	1.60E-09	294484	0.48	1.03 (0.95, 1.12)	0.48	15699	0.48	1.02 (0.95, 1.1)	0.5	15699
rs2642438	1	220970028	1-Mar	G	A	0.7	0.41 (0.34, 0.49)	1.40E-27	294484	0.7	1 (0.98, 1.02)	0.94	395066	0.7	0.98 (0.96, 1)	0.096	394846
rs17713879	2	254215	SH3YL1	A	G	0.37	0.22 (0.15, 0.29)	1.30E-09	294484	0.35	0.98 (0.97, 1)	0.11	395066	0.35	1 (0.98, 1.02)	0.89	394846
rs3820897	2	3642361	COLEC11	T	C	0.18	0.35 (0.26, 0.44)	1.70E-14	294484	0.19	1.01 (0.98, 1.03)	0.62	395066	0.19	1.01 (0.99, 1.03)	0.41	394846
rs1107850	2	20371772	SDC1	G	A	0.53	0.3 (0.23, 0.37)	1.10E-17	294484	0.53	0.99 (0.98, 1.01)	0.44	395066	0.53	0.99 (0.97, 1.01)	0.31	394846
rs2678379	2	21226560	APOB	A	G	0.2	0.88 (0.8, 0.97)	1.10E-93	294484	0.22	1 (0.98, 1.02)	0.9	410811	0.22	1 (0.98, 1.02)	0.74	410591
rs62132319	2	44857962	CAMKMT	T	G	0.35	0.21 (0.14, 0.29)	4.90E-09	294484	0.36	1 (0.98, 1.01)	0.63	395066	0.36	1 (0.98, 1.02)	0.92	394846
rs4599108	2	85543222	TGOLN2	T	C	0.48	0.21 (0.15, 0.28)	1.10E-09	294484	0.49	0.99 (0.97, 1.01)	0.19	395066	0.49	0.99 (0.98, 1.01)	0.56	394846
rs7562358	2	128587317	POLR2D	C	T	0.52	0.21 (0.14, 0.28)	1.50E-09	294484	0.52	1.01 (1, 1.03)	0.098	410764	0.52	1 (0.98, 1.02)	0.84	410544
rs79953491	2	165555539	COBLL1	G	A	0.12	0.57 (0.46, 0.67)	3.50E-26	294484	0.12	0.97 (0.95, 1)	0.073	395066	0.12	1.02 (0.99, 1.05)	0.21	394846
rs1047891	2	211540507	CPS1	C	A	0.68	0.27 (0.2, 0.35)	2.50E-13	294484	0.68	0.99 (0.91, 1.09)	0.91	15697	0.68	0.94 (0.87, 1.01)	0.11	15697
rs59104589	2	242237902	HDLBP	T	C	0.36	0.23 (0.16, 0.31)	8.90E-11	294484	0.35	1 (0.99, 1.02)	0.7	410728	0.35	1 (0.98, 1.02)	0.82	410508
rs12485478	3	12351223	PPARG	A	G	0.97	0.82 (0.61, 1.04)	6.30E-14	294484	0.97	1.03 (0.97, 1.08)	0.33	410813	0.97	0.99 (0.94, 1.04)	0.66	410593
rs13097947	3	15846011	ANKRD28	C	T	0.65	0.26 (0.19, 0.33)	1.70E-12	294484	0.65	0.97 (0.95, 0.99)	0.0056	395066	0.65	1 (0.98, 1.02)	0.98	394846
rs113966471	3	52478802	SEMA3G	T	C	0.06	0.62 (0.47, 0.76)	3.40E-16	294484	0.06	1.07 (1.03, 1.11)	0.00025	410648	0.06	1.05 (1.01, 1.09)	0.018	410428
rs3732356	3	119529113	NR1I2	G	T	0.07	0.48 (0.35, 0.62)	7.40E-12	294484	0.08	1.04 (1, 1.08)	0.031	395066	0.08	1.04 (1, 1.08)	0.043	394846
rs6762415	3	133478557	TF	T	G	0.47	0.24 (0.17, 0.31)	3.50E-12	294484	0.47	1 (0.98, 1.01)	0.61	395066	0.47	0.99 (0.98, 1.01)	0.56	394846

rs866782	3	135983497	PCCB	T	C	0.2	0.34 (0.25, 0.42)	5.40E-15	294484	0.2	0.98 (0.95, 1)	0.026	395066	0.2	0.95 (0.93, 0.98)	3.60E-05	394846
rs10513801	3	185822353	ETV5	T	G	0.86	0.47 (0.37, 0.57)	3.40E-20	294484	0.87	1.04 (1.01, 1.07)	0.0072	410776	0.87	1.04 (1.02, 1.07)	0.0015	410556
rs12510382	4	970112	DGKQ	G	A	0.46	0.25 (0.18, 0.32)	1.00E-12	294484	0.45	1 (0.98, 1.02)	0.95	395066	0.45	1 (0.98, 1.02)	0.89	394846
rs767617	4	39696766	UBE2K	T	C	0.5	0.2 (0.13, 0.27)	5.80E-09	294484	0.52	1.01 (0.93, 1.1)	0.81	15684	-	-	-	-
rs2175766	4	76572191	G3BP2	C	A	0.54	0.19 (0.12, 0.26)	3.20E-08	294484	0.53	1.01 (0.99, 1.03)	0.39	410813	0.53	1 (0.98, 1.01)	0.58	410593
rs2167750	4	89730074	FAM13A	C	T	0.54	0.3 (0.23, 0.36)	1.70E-17	294484	0.53	0.98 (0.97, 1)	0.065	395066	0.53	1 (0.98, 1.01)	0.72	394846
rs11729869	4	120254961	FABP2	C	T	0.58	0.2 (0.13, 0.28)	2.70E-08	294484	0.62	0.99 (0.97, 1.01)	0.51	395066	0.62	1.01 (0.99, 1.03)	0.57	394846
rs13134992	4	146393096	SMAD1	T	C	0.59	0.2 (0.13, 0.27)	9.70E-09	294484	0.57	1.01 (1, 1.03)	0.13	395066	0.57	1.01 (0.99, 1.03)	0.25	394846
rs7725218	5	1282414	TERT	G	A	0.66	0.21 (0.14, 0.29)	6.30E-09	294484	0.66	1 (0.98, 1.02)	0.83	395066	0.66	1 (0.98, 1.02)	0.86	394846
rs435234	5	16641948	MYO10	T	C	0.23	0.23 (0.15, 0.32)	1.30E-08	294484	0.22	1.01 (0.99, 1.03)	0.42	395066	0.22	0.99 (0.97, 1.02)	0.62	394846
rs7730268	5	43824263	-	T	G	0.92	0.4 (0.27, 0.53)	8.40E-10	294484	0.93	0.97 (0.94, 1.01)	0.11	410711	0.93	0.99 (0.96, 1.03)	0.67	410491
rs2307111	5	75003678	POC5	C	T	0.39	0.3 (0.23, 0.37)	9.70E-17	294484	0.4	1 (0.98, 1.02)	0.92	410752	0.4	0.99 (0.97, 1)	0.15	410532
rs1389849	5	111239445	NREP	T	C	0.68	0.21 (0.14, 0.28)	1.20E-08	294484	0.66	0.97 (0.89, 1.06)	0.47	15727	0.66	1.05 (0.97, 1.13)	0.2	15727
rs3812049	5	127418850	SLC12A2	C	G	0.26	0.23 (0.15, 0.31)	6.70E-09	294484	0.26	1 (0.98, 1.02)	0.96	395066	0.26	1.03 (1.01, 1.05)	0.0087	394846
rs188238483	5	180308441	BTNL8	T	C	0.99	0.95 (0.62, 1.28)	1.60E-08	294484	0.99	1.05 (0.95, 1.15)	0.33	395066	0.99	0.93 (0.85, 1.03)	0.16	394846
rs9505097	6	7255650	RREB1	T	C	0.2	0.25 (0.16, 0.33)	1.70E-08	294484	0.19	1 (0.97, 1.02)	0.68	395066	0.19	0.99 (0.97, 1.02)	0.62	394846
rs1233380	6	29603582	GABBR1	C	G	0.65	0.29 (0.22, 0.36)	5.60E-15	294484	0.67	1.02 (1, 1.04)	0.014	395066	0.67	1.01 (0.99, 1.03)	0.45	394846
rs9471972	6	42915021	CNPY3	A	G	0.54	0.3 (0.23, 0.37)	9.30E-18	294484	0.53	1 (0.98, 1.02)	0.8	395066	0.53	1 (0.98, 1.02)	0.89	394846
rs9401593	6	98549801	-	C	A	0.49	0.2 (0.13, 0.27)	8.20E-09	294484	0.48	0.99 (0.97, 1.01)	0.27	395066	0.48	1 (0.98, 1.02)	0.88	394846
rs2800710	6	127452581	RSPO3	T	C	0.48	0.29 (0.22, 0.36)	3.50E-17	294484	0.49	1.01 (0.99, 1.02)	0.55	410814	0.49	0.99 (0.97, 1.01)	0.24	410594
rs6570299	6	139340944	ABRACL	T	A	0.48	0.19 (0.12, 0.26)	5.00E-08	294484	0.48	1.02 (1, 1.04)	0.052	410783	0.48	1.02 (1, 1.03)	0.091	410563
rs634869	6	139831757	-	C	T	0.59	0.28 (0.21, 0.35)	8.60E-16	294484	0.58	1.01 (1, 1.03)	0.13	410814	0.58	1 (0.99, 1.02)	0.61	410594
rs62428831	6	143247161	HIVEP2	C	T	0.14	0.28 (0.18, 0.38)	3.50E-08	294484	0.13	0.97 (0.95, 1)	0.052	395066	0.13	1.03 (1, 1.06)	0.046	394846
rs41272086	6	161008646	LPA	G	A	0.89	0.86 (0.75, 0.97)	1.60E-52	294484	0.9	1.03 (1, 1.06)	0.049	395066	0.9	1 (0.97, 1.03)	0.82	394846
rs9306	6	163736204	PACRG	T	C	0.58	0.21 (0.14, 0.28)	2.40E-09	294484	-	-	-	-	0.58	0.95 (0.88, 1.02)	0.14	15689
rs10233430	7	1051664	C7orf50	T	C	0.57	0.33 (0.26, 0.4)	4.40E-21	294484	0.58	0.99 (0.97, 1.01)	0.23	410800	0.58	1.02 (1, 1.04)	0.025	410580
rs79949326	7	6461310	DAGLB	T	C	0.26	0.36 (0.28, 0.43)	1.80E-19	294484	0.25	1.01 (0.99, 1.03)	0.48	395066	0.25	0.99 (0.97, 1.01)	0.2	394846
rs17138358	7	17920253	SNX13	G	C	0.6	0.4 (0.33, 0.47)	5.30E-29	294484	0.6	1.01 (0.99, 1.03)	0.23	395066	0.6	1 (0.98, 1.02)	0.74	394846
rs11185603	7	50306810	-	G	C	0.32	0.27 (0.2, 0.35)	2.80E-13	294484	0.32	1 (0.98, 1.02)	0.91	410791	0.32	1 (0.98, 1.02)	0.94	410571
rs35493868	7	73039406	MLXIPL	G	C	0.2	0.52 (0.43, 0.6)	4.90E-33	294484	0.19	1 (0.98, 1.02)	0.96	410792	0.19	1.01 (0.99, 1.03)	0.41	410572
rs183906992	7	95011948	PON1; PON3	C	T	0.04	0.58 (0.41, 0.75)	2.00E-11	294484	0.04	1 (0.95, 1.05)	0.94	395066	0.04	1.02 (0.97, 1.07)	0.46	394846
rs909152	7	100175473	LRCH4	T	C	0.33	0.21 (0.14, 0.28)	9.50E-09	294484	0.31	1.02 (1, 1.04)	0.086	395066	0.31	1.01 (0.99, 1.03)	0.51	394846
rs6977416	7	150542711	AOC1	G	A	0.67	0.26 (0.19, 0.33)	1.90E-12	294484	0.66	1.01 (1, 1.03)	0.13	410805	0.66	1 (0.98, 1.02)	0.73	410585
rs10088815	8	34387585	-	G	A	0.4	0.21 (0.14, 0.28)	2.10E-09	294484	0.39	1 (0.98, 1.01)	0.64	395066	0.39	0.99 (0.97, 1.01)	0.24	394846

rs1425604	8	64796303	-	A	C	0.47	0.21 (0.14, 0.27)	2.80E-09	294484	0.47	0.98 (0.96, 1)	0.015	395066	0.47	0.98 (0.96, 1)	0.023	394846
rs10504477	8	71338185	NCOA2	T	C	0.59	0.23 (0.16, 0.3)	9.30E-11	294484	0.59	0.99 (0.91, 1.08)	0.89	15641	0.59	1.05 (0.97, 1.12)	0.24	15641
rs35756394	8	72419796	RP11-1102P16.1	A	G	0.92	0.37 (0.25, 0.5)	4.60E-09	294484	0.93	1 (0.97, 1.04)	0.82	410772	0.93	1.02 (0.99, 1.05)	0.26	410552
rs2513877	8	103883630	AZIN1; KB-1507C5.2	A	G	0.19	0.27 (0.18, 0.35)	2.40E-09	294484	0.19	0.99 (0.97, 1.01)	0.25	410732	0.19	0.98 (0.96, 1.01)	0.13	410512
rs13263073	8	105982216	RP11-127H5.1	A	C	0.81	0.3 (0.21, 0.39)	5.10E-11	294484	0.8	1.03 (0.93, 1.14)	0.59	15598	0.8	0.97 (0.89, 1.06)	0.54	15598
rs10955991	8	121867780	-	T	C	0.32	0.29 (0.21, 0.36)	1.80E-14	294484	0.32	0.96 (0.88, 1.05)	0.33	15668	0.32	0.98 (0.91, 1.06)	0.57	15668
rs4871603	8	126480367	TRIB1	T	C	0.66	0.52 (0.45, 0.6)	1.10E-46	294484	0.64	0.99 (0.97, 1.01)	0.22	410804	0.64	0.99 (0.97, 1.01)	0.35	410584
rs7832515	8	144306379	GPIHBP1	G	A	0.19	0.57 (0.48, 0.65)	1.50E-36	294484	0.19	1 (0.97, 1.02)	0.8	395066	0.19	1 (0.97, 1.02)	0.78	394846
rs1396636	9	1040986	DMRT2	G	A	0.84	0.26 (0.17, 0.35)	4.30E-08	294484	0.84	0.99 (0.96, 1.01)	0.33	395066	0.84	1.01 (0.99, 1.04)	0.31	394846
rs686030	9	15304782	TTC39B	A	C	0.86	0.75 (0.66, 0.85)	8.10E-52	294484	0.85	1.01 (0.98, 1.03)	0.54	395066	0.85	0.98 (0.95, 1)	0.054	394846
rs12351518	9	19357812	DENND4C	C	G	0.1	0.38 (0.27, 0.49)	1.90E-11	294484	0.11	1.01 (0.98, 1.04)	0.55	395066	0.11	1.02 (0.99, 1.05)	0.29	394846
rs296886	9	86592026	HNRNPK	G	A	0.21	0.24 (0.16, 0.33)	9.00E-09	294484	0.21	1.01 (0.99, 1.03)	0.44	395066	0.21	1 (0.98, 1.02)	0.94	394846
rs76530346	9	94010298	AUH	G	A	0.9	0.35 (0.24, 0.47)	2.60E-09	294484	0.91	0.99 (0.96, 1.03)	0.71	395066	0.91	1.03 (1, 1.07)	0.043	394846
rs4979371	9	117138278	AKNA	T	C	0.49	0.23 (0.17, 0.3)	2.10E-11	294484	0.49	0.98 (0.96, 1)	0.034	395066	0.49	1.01 (0.99, 1.03)	0.17	394846
rs2417125	9	131562232	TBC1D13	A	G	0.72	0.22 (0.14, 0.29)	1.70E-08	294484	0.72	1 (0.98, 1.02)	0.71	395066	0.72	1.01 (0.99, 1.03)	0.56	394846
rs11254464	10	17265447	VIM	C	T	0.42	0.23 (0.16, 0.29)	1.50E-10	294484	0.43	0.99 (0.97, 1.01)	0.23	395066	0.43	1 (0.98, 1.01)	0.59	394846
rs55665473	10	46022005	8-Mar	A	G	0.24	0.42 (0.34, 0.5)	2.00E-25	294484	0.24	0.99 (0.97, 1.01)	0.26	410804	0.24	1.01 (0.99, 1.03)	0.51	410584
rs10761737	10	65052205	JMJD1C	C	T	0.42	0.25 (0.18, 0.32)	1.00E-12	294484	0.41	1.02 (1, 1.03)	0.087	410806	0.41	1 (0.99, 1.02)	0.64	410586
rs703966	10	80954251	ZMIZ1	A	G	0.42	0.22 (0.15, 0.29)	2.20E-10	294484	0.4	0.98 (0.96, 0.99)	0.0072	395066	0.4	1 (0.98, 1.02)	0.76	394846
rs11202156	10	88511326	BMPR1A	G	T	0.73	0.23 (0.15, 0.3)	7.70E-09	294484	0.72	1 (0.98, 1.02)	0.92	395066	0.72	1 (0.98, 1.02)	0.65	394846
rs2792751	10	113940329	GPAM	T	C	0.27	0.54 (0.47, 0.62)	4.50E-44	294484	0.29	0.98 (0.96, 1)	0.08	395066	0.29	1 (0.98, 1.01)	0.63	394846
rs7935908	11	567630	RASSF7	G	A	0.38	0.2 (0.13, 0.27)	3.90E-08	294484	0.39	1.02 (1, 1.04)	0.064	395066	0.39	0.99 (0.98, 1.01)	0.56	394846
rs16928809	11	2936952	SLC22A18	G	A	0.91	0.44 (0.32, 0.56)	2.90E-13	294484	0.9	1.02 (0.99, 1.06)	0.16	410793	0.9	1 (0.97, 1.04)	0.84	410573
rs17309930	11	27748493	BDNF	C	A	0.79	0.31 (0.23, 0.39)	4.30E-13	294484	0.8	0.97 (0.95, 0.99)	0.0058	395066	0.8	0.97 (0.95, 1)	0.018	394846
rs7943729	11	45183379	PRDM11	C	T	0.88	0.31 (0.2, 0.41)	1.30E-08	294484	0.88	1.01 (0.98, 1.04)	0.6	395066	0.88	1.01 (0.98, 1.04)	0.51	394846
rs174566	11	61592362	FADS2; FADS1	A	G	0.65	0.87 (0.8, 0.94)	4.40E-126	294484	0.66	1.02 (1, 1.03)	0.088	410814	0.66	1.03 (1.01, 1.05)	0.00099	410594
rs56271783	11	64004723	VEGFB	G	C	0.95	0.69 (0.53, 0.86)	2.10E-16	294484	0.95	0.98 (0.94, 1.02)	0.42	395066	0.95	1.01 (0.97, 1.05)	0.7	394846
rs4930352	11	66066993	TMEM151A	T	G	0.5	0.25 (0.18, 0.32)	3.00E-12	294484	0.48	1 (0.98, 1.02)	0.98	395066	0.48	1 (0.98, 1.01)	0.65	394846
rs11021232	11	95320808	-	T	C	0.82	0.29 (0.2, 0.38)	1.50E-10	294484	0.82	0.97 (0.95, 0.99)	0.015	395066	0.82	1 (0.97, 1.02)	0.68	394846
rs12146566	11	103871404	PDGFD	A	C	0.8	0.24 (0.16, 0.33)	2.60E-08	294484	0.8	1.03 (0.93, 1.14)	0.58	15733	0.8	0.96 (0.88, 1.05)	0.37	15733
rs1177562	11	118949331	VPS11	C	T	0.59	0.29 (0.22, 0.36)	4.40E-16	294484	0.59	0.98 (0.96, 0.99)	0.0072	410763	0.59	0.99 (0.97, 1)	0.11	410543
rs1622638	11	121800971	-	G	A	0.61	0.22 (0.15, 0.28)	1.40E-09	294484	0.6	1 (0.99, 1.02)	0.61	395066	0.6	0.99 (0.97, 1.01)	0.18	394846
rs58473820	11	122514403	UBASH3B	T	C	0.38	0.42 (0.35, 0.49)	3.20E-31	294484	0.37	1.02 (1, 1.03)	0.091	395066	0.37	1.01 (0.99, 1.03)	0.25	394846

rs7298463	12	6663964	IFFO1	G	C	0.83	0.31 (0.22, 0.4)	2.90E-11	294484	0.83	0.98 (0.96, 1.01)	0.13	395066	0.83	0.97 (0.95, 1)	0.043	394846
rs11045172	12	20470221	PDE3A	C	A	0.2	0.44 (0.36, 0.53)	3.60E-24	294484	0.2	1 (0.98, 1.03)	0.7	395066	0.2	1 (0.98, 1.03)	0.79	394846
rs7305031	12	27020853	ITPR2	T	C	0.07	0.39 (0.26, 0.52)	1.40E-08	294484	0.06	1 (0.96, 1.03)	0.84	395066	0.06	0.97 (0.93, 1)	0.072	394846
rs4332553	12	56469953	ERBB3	T	C	0.59	0.21 (0.14, 0.28)	4.50E-09	294484	0.58	0.98 (0.96, 1)	0.021	395066	0.58	1 (0.98, 1.01)	0.65	394846
rs61352607	12	57839173	INHBC	T	G	0.24	0.47 (0.4, 0.55)	5.90E-32	294484	0.23	0.99 (0.97, 1.01)	0.37	410806	0.23	1.03 (1.01, 1.05)	0.002	410586
rs10861632	12	106960182	RP11-144F15.1	G	A	0.74	0.25 (0.17, 0.32)	1.10E-09	294484	0.75	0.98 (0.89, 1.08)	0.73	15666	0.75	1.04 (0.95, 1.13)	0.41	15666
rs7308864	12	109871179	MYO1H	G	A	0.52	0.39 (0.32, 0.45)	8.20E-29	294484	0.53	1.02 (1, 1.04)	0.037	410811	0.53	1.01 (0.99, 1.03)	0.34	410591
rs3184504	12	111884608	SH2B3	C	T	0.52	0.34 (0.28, 0.41)	4.10E-23	294484	0.51	0.97 (0.96, 0.99)	0.0019	395066	0.51	0.98 (0.96, 1)	0.015	394846
rs7970695	12	121423376	HNF1A	G	A	0.38	0.26 (0.19, 0.33)	9.00E-13	294484	0.4	1.04 (1.02, 1.06)	4.40E-05	410721	0.4	1 (0.99, 1.02)	0.69	410501
rs117230571	13	41689067	WBP4	A	G	0.92	0.36 (0.23, 0.49)	3.70E-08	294484	0.92	1 (0.96, 1.03)	0.78	395066	0.92	0.99 (0.95, 1.02)	0.42	394846
rs866376	13	47230865	LRCH1	C	A	0.75	0.22 (0.14, 0.3)	4.50E-08	294484	0.68	1.01 (0.99, 1.03)	0.5	395066	0.68	1.03 (1.01, 1.05)	0.0089	394846
rs9591325	13	50811220	DLEU1	C	T	0.07	0.38 (0.25, 0.52)	2.80E-08	294484	0.07	1.01 (0.97, 1.04)	0.73	410709	0.07	0.97 (0.94, 1)	0.087	410489
rs10145740	14	74234294	ELMSAN1	T	C	0.24	0.27 (0.19, 0.35)	2.00E-11	294484	0.25	1.01 (0.98, 1.03)	0.59	395066	0.25	1 (0.98, 1.02)	0.87	394846
rs2005864	14	75377692	RPS6KL1	C	T	0.56	0.22 (0.16, 0.29)	1.60E-10	294484	0.56	1 (0.98, 1.01)	0.72	395066	0.56	1.01 (0.99, 1.03)	0.32	394846
rs9646167	14	81635888	GTF2A1	C	T	0.48	0.19 (0.12, 0.26)	4.20E-08	294484	0.49	0.99 (0.97, 1.01)	0.43	395066	0.49	1.01 (0.99, 1.03)	0.29	394846
rs12887521	14	103247844	TRAF3	A	C	0.66	0.22 (0.15, 0.29)	9.80E-10	294484	0.65	0.99 (0.97, 1.01)	0.21	395066	0.65	0.98 (0.96, 1)	0.068	394846
rs2494748	14	105258892	AKT1	C	T	0.38	0.4 (0.33, 0.47)	1.50E-29	294484	0.4	0.98 (0.97, 1)	0.076	395066	0.4	0.99 (0.98, 1.01)	0.52	394846
rs150844304	15	43726625	TP53BP1	A	C	0.98	1.34 (1.12, 1.56)	4.50E-33	294484	0.97	1.03 (0.97, 1.09)	0.32	395066	0.97	1.04 (0.98, 1.1)	0.22	394846
rs2131739	15	57917064	GCOM1; MYZAP; POLR2M	C	T	0.76	0.22 (0.14, 0.3)	4.40E-08	294484	0.76	0.99 (0.97, 1.02)	0.63	395066	0.76	1.01 (0.99, 1.03)	0.45	394846
rs3803501	15	63352092	TPM1	A	G	0.57	0.25 (0.18, 0.32)	1.30E-12	294484	0.56	1 (0.99, 1.02)	0.61	395066	0.56	0.98 (0.96, 1)	0.028	394846
rs34675318	15	64167098	-	G	A	0.71	0.22 (0.14, 0.29)	1.10E-08	294484	0.7	1.02 (1, 1.03)	0.12	410787	0.7	0.99 (0.97, 1.01)	0.36	410567
rs28362901	15	74712937	SEMA7A	C	A	0.91	0.4 (0.28, 0.52)	1.00E-10	294484	0.92	0.96 (0.93, 0.99)	0.012	410781	0.92	0.98 (0.95, 1.01)	0.14	410561
rs4238539	15	83464612	FSD2	A	T	0.78	0.24 (0.16, 0.32)	1.60E-08	294484	0.78	0.98 (0.96, 1)	0.097	395066	0.78	0.99 (0.97, 1.02)	0.62	394846
rs4784744	16	57011185	CETP	G	A	0.65	1.35 (1.28, 1.42)	3.00E-299	294484	0.65	0.99 (0.97, 1.01)	0.47	395066	0.65	1.01 (0.99, 1.03)	0.26	394846
rs9926558	16	62892035	-	C	A	0.82	0.26 (0.17, 0.35)	3.80E-08	294484	0.68	1.01 (0.93, 1.11)	0.79	15720	0.68	0.99 (0.91, 1.06)	0.72	15720
rs4788556	16	71793673	AP1G1	T	A	0.84	0.32 (0.22, 0.41)	1.90E-11	294484	0.83	0.99 (0.97, 1.02)	0.6	410781	0.83	1 (0.97, 1.02)	0.76	410561
rs870021	16	88536774	ZFPM1	T	A	0.68	0.21 (0.14, 0.29)	9.00E-09	294484	0.68	1.01 (0.99, 1.03)	0.22	395066	0.68	0.99 (0.97, 1.01)	0.55	394846
rs3803800	17	7462969	TNFSF12; TNFSF12-TNFSF13; TNFSF13	G	A	0.79	0.35 (0.26, 0.43)	3.30E-16	294484	0.79	1 (0.98, 1.02)	0.8	395066	0.79	1 (0.98, 1.02)	1	394846
rs1476161	17	8090908	C17orf59	A	G	0.39	0.21 (0.14, 0.28)	3.30E-09	294484	0.41	1.01 (0.99, 1.03)	0.28	395066	0.41	1 (0.98, 1.02)	0.81	394846
rs75634664	17	17538289	PEMT	C	G	0.94	0.5 (0.35, 0.65)	2.90E-11	294484	0.94	1.04 (1.01, 1.08)	0.02	410757	0.94	0.99 (0.96, 1.03)	0.78	410537
rs10512432	17	28812120	GOSR1	G	A	0.65	0.2 (0.13, 0.27)	2.50E-08	294484	0.67	0.99 (0.97, 1)	0.14	395066	0.67	0.99 (0.97, 1.01)	0.2	394846

rs881844	17	37810218	STARD3	G	C	0.67	0.44 (0.36, 0.51)	2.30E-32	294484	0.65	0.95 (0.93, 0.97)	9.30E-09	410767	0.65	0.98 (0.97, 1)	0.072	410547
rs117499775	17	44078618	MAPT	T	C	0.96	0.49 (0.31, 0.66)	2.80E-08	294484	0.97	0.97 (0.91, 1.02)	0.23	395066	0.97	0.93 (0.88, 0.98)	0.01	394846
rs595767	17	46957987	ATP5G1	A	G	0.48	0.2 (0.13, 0.26)	1.60E-08	294484	0.49	0.98 (0.96, 1)	0.024	395066	0.49	0.98 (0.96, 0.99)	0.0068	394846
rs112001035	17	66823805	ABCA8	G	A	0.94	0.72 (0.58, 0.87)	7.20E-23	294484	0.94	0.97 (0.93, 1.01)	0.16	395066	0.94	0.97 (0.93, 1.01)	0.094	394846
rs7238995	18	20085103	-	A	T	0.53	0.19 (0.12, 0.26)	4.30E-08	294484	0.54	1 (0.98, 1.02)	0.8	410673	0.54	1 (0.99, 1.02)	0.69	410453
rs7245642	19	4053013	ZBTB7A	G	A	0.28	0.27 (0.2, 0.35)	2.50E-12	294484	0.29	0.98 (0.96, 1)	0.036	410737	0.29	0.99 (0.97, 1.01)	0.36	410517
rs2278426	19	11350488	DOCK6; C19orf80	C	T	0.97	1.29 (1.1, 1.48)	1.30E-41	294484	0.96	1.01 (0.97, 1.06)	0.54	410777	0.96	0.99 (0.95, 1.04)	0.81	410557
rs4805881	19	33896432	PEPD	C	A	0.67	0.29 (0.22, 0.36)	2.10E-15	294484	0.66	1 (0.98, 1.02)	0.73	410778	0.66	1.01 (0.99, 1.03)	0.26	410558
rs4802113	19	41740895	AXL	C	T	0.46	0.2 (0.13, 0.27)	9.50E-09	294484	0.44	0.98 (0.97, 1)	0.056	395066	0.44	1.02 (1, 1.04)	0.046	394846
rs4760	19	44153100	PLAUR	A	G	0.84	0.27 (0.17, 0.36)	1.90E-08	294484	0.85	0.99 (0.97, 1.02)	0.62	410747	0.85	1 (0.97, 1.02)	0.81	410527
rs429358	19	45411941	APOE	T	C	0.84	0.87 (0.78, 0.97)	9.40E-75	294484	0.86	1 (0.98, 1.03)	0.73	410667	0.86	1.02 (1, 1.05)	0.08	410447
rs34255979	19	46384830	IRF2BP1	C	T	0.88	0.51 (0.4, 0.61)	3.20E-21	294484	0.88	0.99 (0.97, 1.02)	0.57	410759	0.88	0.98 (0.96, 1.01)	0.2	410539
rs721889	19	52341249	FPR3	A	G	0.66	0.29 (0.21, 0.36)	4.70E-15	294484	0.65	1 (0.99, 1.02)	0.64	395066	0.65	0.97 (0.95, 0.99)	0.0022	394846
rs367070	19	54800500	LILRA3	G	A	0.23	0.62 (0.54, 0.7)	8.20E-51	294484	0.2	1.02 (1, 1.04)	0.11	395066	0.2	1 (0.97, 1.02)	0.73	394846
rs3736802	20	33604042	TRPC4AP	C	T	0.52	0.26 (0.19, 0.32)	2.00E-13	294484	0.53	1.02 (1, 1.03)	0.068	410781	0.53	0.99 (0.97, 1)	0.13	410561
rs4239651	20	46340596	SULF2	C	T	0.79	0.36 (0.28, 0.45)	2.30E-17	294484	0.8	1.01 (0.99, 1.03)	0.35	410746	0.8	1 (0.98, 1.02)	0.9	410526
rs76602912	20	57459868	GNAS	T	C	0.98	0.64 (0.42, 0.87)	1.70E-08	294484	0.98	1.06 (0.99, 1.14)	0.1	395066	0.98	0.98 (0.91, 1.05)	0.5	394846
rs7281183	21	16411667	NRIP1	G	A	0.27	0.22 (0.14, 0.3)	2.90E-08	294484	0.27	0.98 (0.96, 1)	0.11	395066	0.27	0.99 (0.97, 1.02)	0.6	394846
rs235314	21	46271452	PTTG1IP	C	T	0.47	0.26 (0.19, 0.32)	2.00E-13	294484	0.47	0.99 (0.97, 1.01)	0.31	410798	0.47	1 (0.98, 1.02)	0.82	410578
rs9980311	21	46899279	COL18A1	A	G	0.74	0.26 (0.18, 0.34)	2.50E-10	294484	0.73	1.01 (0.99, 1.03)	0.59	395066	0.73	0.99 (0.97, 1.02)	0.6	394846
rs2256609	22	21925017	UBE2L3	A	G	0.81	0.45 (0.36, 0.53)	3.60E-24	294484	0.81	1.03 (0.92, 1.14)	0.63	15743	0.81	1 (0.91, 1.09)	0.96	15743
rs4820324	22	38599857	PLA2G6; MAFF	G	C	0.42	0.27 (0.2, 0.34)	8.50E-15	294484	0.43	1.01 (0.99, 1.03)	0.22	410791	0.43	1.01 (0.99, 1.03)	0.2	410571
rs738409	22	44324727	PNPLA3	C	G	0.78	0.24 (0.15, 0.32)	2.20E-08	294484	0.77	1 (0.98, 1.02)	0.93	410803	0.77	1.01 (0.99, 1.03)	0.49	410583

Chr: Chromosome; EA: Effect Allele; NEA: Non-effect Allele; EAF: Effect Allele Frequency.
SNPs excluded in the MR-PRESSO analysis of HFrEF: rs113966471, rs1771582, rs646776, rs7970695, rs881844.
SNPs excluded in the MR-PRESSO analysis of HFpEF: rs866782.

Table S6. GWAS Summary Used for Mendelian Randomization Analysis of Low-density Lipoprotein Cholesterol (LDL-C).

rsID	Chr	Position (hg19)	Gene	EA	NEA	LDL-C				HFrEF				HFpEF			
						EAF	Beta (95% CI)	P	N	EAF	OR (95% CI)	P	N	EAF	OR (95% CI)	P	N
rs61778886	1	23795181	ASAP3	C	G	0.87	0.69 (0.47, 0.91)	1.50E-09	321090	0.86	1.02 (0.99, 1.05)	0.15	395066	0.86	1.01 (0.98, 1.03)	0.71	394846
rs1556562	1	93034023	EVI5	T	G	0.79	0.67 (0.5, 0.85)	1.10E-13	321090	0.79	1.1 (0.99, 1.22)	0.065	15640	0.79	0.96 (0.88, 1.05)	0.4	15640
rs6657811	1	109807283	CELSR2	A	T	0.87	3.97 (3.75, 4.19)	5.50E-268	321090	0.88	1.05 (1.02, 1.08)	0.00044	395066	0.88	1.04 (1.02, 1.07)	0.0022	394846
rs267733	1	150958836	ANXA9	A	G	0.84	0.7 (0.5, 0.91)	1.10E-11	321090	0.84	1.01 (0.98, 1.03)	0.5	410797	0.84	1.01 (0.98, 1.03)	0.63	410577
rs4440846	1	155130798	DPM3	T	C	0.17	0.57 (0.37, 0.78)	3.50E-08	321090	0.17	0.99 (0.97, 1.02)	0.49	395066	0.17	1.01 (0.98, 1.03)	0.68	394846
rs144126567	1	161510516	FCGR3A	G	C	0.08	0.82 (0.53, 1.1)	2.30E-08	321090	0.08	1.04 (1.01, 1.08)	0.0084	395066	0.08	0.98 (0.95, 1.01)	0.21	394846
rs10912854	1	174898715	RABGAP1L	C	G	0.27	0.49 (0.32, 0.66)	1.40E-08	321090	0.27	1 (0.98, 1.02)	0.79	410734	0.27	1 (0.98, 1.02)	0.9	410514
rs2807834	1	220970593	1-Mar	G	T	0.69	0.91 (0.75, 1.07)	2.40E-28	321090	0.68	1 (0.98, 1.02)	0.87	410645	0.68	0.99 (0.97, 1.01)	0.34	410425
rs35804313	1	224667102	CNIH3	C	T	0.78	0.55 (0.37, 0.73)	3.40E-09	321090	0.78	1.02 (1, 1.05)	0.044	395066	0.78	1.01 (0.99, 1.03)	0.52	394846
rs556107	1	234853059	IRF2BP2	T	C	0.52	1.43 (1.28, 1.58)	3.50E-78	321090	0.53	1 (0.92, 1.08)	0.94	15653	0.53	1.03 (0.96, 1.1)	0.48	15653
rs6542680	2	3640142	COLEC11	C	T	0.18	0.63 (0.44, 0.83)	2.40E-10	321090	0.19	1.01 (0.98, 1.03)	0.61	395066	0.19	1.01 (0.99, 1.03)	0.37	394846
rs11673790	2	21299195	APOB	T	C	0.33	3.12 (2.96, 3.28)	4.9e-324	321090	0.31	1 (0.99, 1.02)	0.62	410814	0.31	0.99 (0.97, 1.01)	0.27	410594
rs78711174	2	24496531	ITSN2	A	G	0.27	0.56 (0.39, 0.73)	6.90E-11	321090	0.25	0.98 (0.96, 1)	0.083	395066	0.25	0.99 (0.97, 1.01)	0.42	394846
rs1260326	2	27730940	GCKR	T	C	0.39	1.6 (1.44, 1.75)	4.70E-93	321090	0.41	0.99 (0.97, 1.01)	0.25	395066	0.41	1 (0.98, 1.02)	0.98	394846
rs4299376	2	44072576	ABCG8	G	T	0.32	2.29 (2.13, 2.44)	6.40E-173	321090	0.33	1.01 (1, 1.03)	0.13	410744	0.33	1.02 (1, 1.04)	0.019	410524
rs687914	2	45878760	PRKCE	T	G	0.25	0.49 (0.32, 0.66)	2.80E-08	321090	0.25	1.03 (1.01, 1.05)	0.0092	395066	0.25	1.02 (1, 1.04)	0.099	394846
rs1861398	2	64906800	SERTAD2	C	G	0.74	0.61 (0.44, 0.78)	3.50E-12	321090	0.72	0.99 (0.97, 1.01)	0.25	410772	0.72	0.99 (0.97, 1.01)	0.39	410552
rs150474434	2	118845121	INSIG2	G	A	0.9	1.35 (1.1, 1.6)	2.60E-26	321090	0.9	1.04 (1.01, 1.07)	0.017	395066	0.9	0.98 (0.95, 1.01)	0.19	394846
rs17050272	2	121306440	-	G	A	0.59	0.78 (0.63, 0.93)	9.00E-24	321090	0.58	1 (0.98, 1.02)	0.98	395066	0.58	1 (0.98, 1.02)	0.83	394846
rs10164853	2	158481992	ACVR1C	A	G	0.92	0.91 (0.62, 1.19)	4.70E-10	321090	0.92	1.02 (0.98, 1.06)	0.29	395066	0.92	0.99 (0.96, 1.03)	0.64	394846
rs13076933	3	12327431	PPARG	T	G	0.74	0.88 (0.71, 1.05)	1.00E-23	321090	0.75	1 (0.98, 1.02)	0.78	395066	0.75	0.98 (0.96, 1.01)	0.15	394846
rs9834932	3	32535382	CMTM6	A	G	0.91	1.28 (1.02, 1.54)	1.30E-21	321090	0.91	1.02 (0.99, 1.06)	0.13	410736	0.91	1.01 (0.98, 1.05)	0.36	410516
rs9844432	3	58391293	PXK	C	G	0.92	1.29 (1.02, 1.56)	1.50E-20	321090	0.92	1.02 (0.99, 1.06)	0.13	410811	0.92	1.03 (1, 1.06)	0.054	410591
rs334558	3	119813282	GSK3B	G	A	0.34	0.58 (0.42, 0.74)	7.80E-13	321090	0.35	1 (0.98, 1.02)	0.84	410766	0.35	0.99 (0.97, 1)	0.14	410546
rs6764238	3	129092491	EFCAB12	C	T	0.1	0.71 (0.46, 0.97)	2.30E-08	321090	0.12	1.01 (0.98, 1.05)	0.46	395066	0.12	1.05 (1.02, 1.08)	0.0031	394846
rs113177823	3	132217703	DNAJC13	G	A	0.95	1.4 (1.07, 1.73)	2.00E-16	321090	0.95	0.96 (0.92, 1)	0.056	395066	0.95	0.98 (0.94, 1.02)	0.41	394846
rs71630059	3	136581611	NCK1	G	A	0.79	0.64 (0.45, 0.82)	8.80E-12	321090	0.8	1.02 (1, 1.04)	0.059	395066	0.8	1.04 (1.02, 1.06)	0.00077	394846
rs11709868	3	142648844	PAQR9	G	T	0.7	0.65 (0.48, 0.81)	1.20E-14	321090	0.71	1 (0.98, 1.02)	0.9	410727	0.71	1 (0.98, 1.02)	0.95	410507
rs4689653	4	7223319	SORCS2	G	T	0.62	0.5 (0.35, 0.65)	2.10E-10	321090	0.61	0.98 (0.97, 1)	0.066	395066	0.61	0.99 (0.98, 1.01)	0.48	394846
rs3775228	4	87985166	AFF1	T	C	0.4	0.54 (0.39, 0.69)	5.00E-12	321090	0.39	0.99 (0.97, 1.01)	0.44	395066	0.39	0.99 (0.97, 1)	0.15	394846

rs35830965	4	120141320	USP53	G	A	0.87	0.68 (0.45, 0.9)	3.40E-09	321090	0.87	1 (0.97, 1.03)	0.97	395066	0.87	1.01 (0.98, 1.04)	0.42	394846
rs116692022	4	155448758	PLRG1	G	A	0.01	2.29 (1.62, 2.97)	2.60E-11	321090	0.01	1.02 (0.92, 1.13)	0.76	410749	0.01	0.96 (0.86, 1.06)	0.4	410529
rs138367557	5	52075451	ITGA1	T	C	0.96	2.01 (1.61, 2.41)	1.10E-22	321090	0.97	1.05 (1, 1.11)	0.041	410812	0.97	0.98 (0.93, 1.03)	0.33	410592
rs9686661	5	55861786	AC022431.2	T	C	0.2	0.81 (0.62, 1)	1.60E-17	321090	0.2	1 (0.98, 1.02)	0.99	410808	0.2	0.99 (0.97, 1.01)	0.27	410588
rs2925677	5	71953629	ZNF366	C	G	0.79	0.57 (0.39, 0.75)	8.00E-10	321090	0.8	1.01 (0.99, 1.03)	0.41	410804	0.8	0.99 (0.97, 1.02)	0.64	410584
rs12916	5	74656539	HMGCR	C	T	0.4	2.49 (2.33, 2.64)	1.20E-224	321090	0.4	1 (0.99, 1.02)	0.64	410808	0.4	1 (0.98, 1.02)	0.88	410588
rs11241702	5	122902603	CSNK1G3	G	A	0.54	0.73 (0.58, 0.88)	1.10E-21	321090	0.54	0.99 (0.97, 1.01)	0.22	410762	0.54	0.97 (0.96, 0.99)	0.0019	410542
rs272838	5	131638817	SLC22A4	C	T	0.84	0.72 (0.52, 0.93)	3.10E-12	321090	0.82	0.97 (0.95, 1)	0.023	395066	0.82	1.01 (0.99, 1.04)	0.25	394846
rs7746081	6	16126934	MYLIP	G	A	0.7	1.09 (0.92, 1.25)	4.50E-39	321090	0.68	1 (0.98, 1.02)	0.88	395066	0.68	1 (0.98, 1.01)	0.62	394846
rs1800562	6	26093141	HFE	G	A	0.92	2.04 (1.77, 2.32)	8.40E-47	321090	0.94	1.02 (0.99, 1.06)	0.2	410811	0.94	1.03 (0.99, 1.06)	0.13	410591
rs4711589	6	39230571	KCNK17	C	A	0.3	0.56 (0.39, 0.72)	3.40E-11	321090	0.29	1.01 (0.99, 1.02)	0.61	410796	0.29	0.99 (0.97, 1.01)	0.36	410576
rs4714638	6	42917017	CNPY3	A	G	0.47	0.46 (0.31, 0.61)	1.30E-09	321090	0.47	1 (0.98, 1.02)	0.8	395066	0.47	1 (0.98, 1.02)	0.83	394846
rs4711750	6	43757082	VEGFA	A	T	0.5	0.46 (0.31, 0.61)	1.40E-09	321090	0.49	1.02 (1, 1.03)	0.098	395066	0.49	0.99 (0.97, 1.01)	0.29	394846
rs67731163	6	52450349	TRAM2	C	T	0.54	0.44 (0.29, 0.59)	8.70E-09	321090	0.57	1 (0.98, 1.02)	0.77	395066	0.57	0.99 (0.97, 1)	0.12	394846
rs12665537	6	53509452	KLHL31	G	A	0.33	0.54 (0.38, 0.7)	2.10E-11	321090	0.31	1.03 (1.01, 1.05)	0.0071	395066	0.31	0.99 (0.97, 1.01)	0.48	394846
rs17185536	6	100620931	-	C	T	0.75	0.7 (0.53, 0.87)	3.50E-15	321090	0.74	0.99 (0.97, 1.01)	0.25	395066	0.74	0.97 (0.95, 0.99)	0.0013	394846
rs9491697	6	127456122	RSPO3	G	A	0.47	0.56 (0.41, 0.71)	2.60E-13	321090	0.45	1 (0.98, 1.02)	0.87	410814	0.45	1.01 (1, 1.03)	0.1	410594
rs12197047	6	130389211	L3MBTL3	A	G	0.67	0.53 (0.37, 0.69)	8.00E-11	321090	0.66	0.99 (0.97, 1.01)	0.52	395066	0.66	0.99 (0.97, 1.01)	0.2	394846
rs9402685	6	135419688	HBS1L	T	C	0.74	0.69 (0.52, 0.86)	2.00E-15	321090	0.74	1 (0.98, 1.02)	0.84	410712	0.74	0.99 (0.97, 1.01)	0.32	410492
rs118039278	6	160985526	LPA	A	G	0.08	3.71 (3.44, 3.99)	5.80E-154	321090	0.07	1.15 (1.11, 1.19)	8.10E-14	395066	0.07	1.06 (1.03, 1.1)	0.00085	394846
rs869412	7	1074134	C7orf50	T	C	0.77	0.62 (0.44, 0.8)	7.90E-12	321090	0.78	1 (0.98, 1.02)	0.97	395066	0.78	1.02 (0.99, 1.04)	0.17	394846
rs836550	7	6440437	RAC1	G	A	0.41	0.44 (0.28, 0.59)	1.90E-08	321090	0.4	1 (0.98, 1.02)	0.98	395066	0.4	1 (0.98, 1.02)	0.99	394846
rs9639288	7	17432838	AHR	C	A	0.77	0.51 (0.33, 0.69)	2.70E-08	321090	0.79	1 (0.98, 1.02)	0.9	395066	0.79	1.02 (1, 1.04)	0.051	394846
rs73066466	7	21599807	DNAH11	C	A	0.23	1.28 (1.11, 1.46)	7.80E-46	321090	0.21	0.99 (0.97, 1.01)	0.29	410698	0.21	1.01 (0.99, 1.03)	0.37	410478
rs67050321	7	36169203	EEPD1	C	T	0.3	0.58 (0.42, 0.75)	2.60E-12	321090	0.31	1.02 (1, 1.04)	0.075	410665	0.31	1.01 (0.99, 1.03)	0.45	410445
rs2073547	7	44582331	NPC1L1	G	A	0.18	1.51 (1.32, 1.7)	1.60E-53	321090	0.19	1 (0.98, 1.02)	0.89	410771	0.19	0.99 (0.97, 1.02)	0.54	410551
rs799157	7	73020301	MLXIPL	T	C	0.04	1.36 (0.99, 1.73)	3.60E-13	321090	0.05	0.98 (0.92, 1.03)	0.38	410806	0.05	0.98 (0.93, 1.04)	0.51	410586
rs2302429	7	75614777	POR	A	G	0.18	0.71 (0.52, 0.91)	5.00E-13	321090	0.18	0.97 (0.95, 1)	0.019	395066	0.18	1 (0.98, 1.03)	0.84	394846
rs12539997	7	87131423	ABCB1	T	C	0.82	0.62 (0.42, 0.81)	5.00E-10	321090	0.82	1.01 (0.99, 1.03)	0.46	395066	0.82	1 (0.98, 1.02)	0.96	394846
rs10953298	7	100216773	TFR2	C	T	0.76	0.81 (0.63, 0.98)	3.80E-19	321090	0.76	1.02 (1, 1.04)	0.079	395066	0.76	1.01 (0.99, 1.03)	0.46	394846
rs2911987	8	6564576	AGPAT5	G	A	0.68	0.5 (0.34, 0.66)	1.30E-09	321090	0.68	1 (0.98, 1.02)	0.84	410674	0.68	0.99 (0.97, 1.01)	0.43	410454
rs1495741	8	18272881	NAT2	G	A	0.22	0.97 (0.79, 1.15)	5.80E-26	321090	0.23	1.02 (1, 1.04)	0.034	410798	0.23	1.02 (1, 1.04)	0.072	410578
rs13702	8	19824492	LPL	T	C	0.71	0.86 (0.69, 1.02)	3.60E-24	321090	0.71	1.01 (0.99, 1.03)	0.23	410810	0.71	1.03 (1.01, 1.05)	0.00069	410590
rs59328596	8	21928227	DMTN	G	A	0.85	0.77 (0.56, 0.98)	1.00E-12	321090	0.86	1.02 (0.99, 1.04)	0.22	395066	0.86	1.01 (0.99, 1.04)	0.29	394846

rs117139027	8	29024943	KIF13B	G	A	0.98	2.39 (1.81, 2.96)	3.50E-16	321090	0.99	1 (0.9, 1.11)	0.94	395066	0.99	1.01 (0.91, 1.12)	0.9	394846
rs9298506	8	55437524	-	G	A	0.21	0.92 (0.74, 1.11)	6.20E-23	321090	0.19	1.01 (0.99, 1.03)	0.4	410812	0.19	1.03 (1.01, 1.05)	0.01	410592
rs4738684	8	59393273	CYP7A1	A	G	0.34	1.23 (1.07, 1.39)	2.00E-52	321090	0.35	0.99 (0.98, 1.01)	0.61	395066	0.35	1.02 (1, 1.04)	0.099	394846
rs615031	8	61539278	RAB2A	T	A	0.37	0.53 (0.38, 0.68)	1.90E-11	321090	0.38	1.01 (0.99, 1.03)	0.16	395066	0.38	1.03 (1.01, 1.05)	0.00094	394846
rs62509311	8	74907295	LY96	A	T	0.72	0.57 (0.41, 0.74)	1.40E-11	321090	0.74	1 (0.98, 1.02)	0.93	395066	0.74	1.02 (1, 1.04)	0.094	394846
rs2737245	8	116658583	TRPS1	G	T	0.72	0.92 (0.75, 1.08)	3.90E-27	321090	0.74	1 (0.99, 1.02)	0.63	410694	0.74	1.01 (0.99, 1.03)	0.31	410474
rs28601761	8	126500031	TRIB1	C	G	0.58	2.66 (2.5, 2.81)	8.70E-254	321090	0.59	1.03 (1.01, 1.04)	0.0059	395066	0.59	1.01 (0.99, 1.03)	0.35	394846
rs3780181	9	2640759	VLDLR	A	G	0.93	1.16 (0.86, 1.46)	4.50E-14	321090	0.93	0.99 (0.96, 1.03)	0.71	410802	0.93	1.01 (0.97, 1.04)	0.62	410582
rs34150222	9	19313913	DENND4C	G	A	0.08	1.13 (0.85, 1.41)	2.00E-15	321090	0.08	1.01 (0.98, 1.04)	0.5	410735	0.08	1.02 (0.99, 1.05)	0.23	410515
rs13301187	9	78068052	-	A	C	0.18	0.6 (0.4, 0.79)	2.10E-09	321090	0.18	1 (0.98, 1.03)	0.83	395066	0.18	0.99 (0.96, 1.01)	0.34	394846
rs1571790	9	78729176	PCSK5	A	T	0.39	0.49 (0.34, 0.65)	3.40E-10	321090	0.4	1 (0.92, 1.09)	0.92	15705	0.4	0.94 (0.87, 1.01)	0.088	15705
rs13289095	9	131466489	PKN3	G	T	0.85	0.81 (0.6, 1.03)	5.60E-14	321090	0.85	1 (0.97, 1.02)	0.93	395066	0.85	1 (0.97, 1.02)	0.95	394846
rs2519093	9	136141870	SURF6	T	C	0.19	2.4 (2.21, 2.59)	1.10E-131	321090	0.19	1.05 (1.03, 1.08)	3.70E-06	410801	0.19	1.03 (1.01, 1.05)	0.015	410581
rs3812591	9	139341612	SEC16A	T	C	0.72	0.53 (0.36, 0.69)	7.20E-10	321090	0.73	1.02 (1, 1.04)	0.081	410798	0.73	1 (0.98, 1.02)	0.84	410578
rs1757218	10	18506741	CACNB2	C	G	0.85	0.63 (0.41, 0.84)	8.60E-09	321090	0.84	1.02 (0.99, 1.04)	0.17	395066	0.84	1 (0.97, 1.02)	0.9	394846
rs16926246	10	71093392	HK1	C	T	0.87	0.9 (0.68, 1.12)	1.60E-15	321090	0.87	0.98 (0.96, 1.01)	0.14	410761	0.87	1 (0.97, 1.03)	0.98	410541
rs1336455	10	97838474	CCNJ	G	A	0.64	0.45 (0.3, 0.61)	1.20E-08	321090	0.64	0.96 (0.88, 1.04)	0.34	15672	0.64	0.98 (0.91, 1.06)	0.64	15672
rs2792735	10	113921825	GPAM	G	A	0.28	0.74 (0.57, 0.9)	4.80E-18	321090	0.31	0.98 (0.96, 1)	0.039	410733	0.31	1 (0.98, 1.02)	0.94	410513
rs2301179	10	118404620	C10orf82	G	A	0.5	0.51 (0.36, 0.66)	3.40E-11	321090	0.5	1 (0.99, 1.02)	0.67	410808	0.5	1.01 (0.99, 1.03)	0.4	410588
rs12246352	10	124705307	C10orf88	G	A	0.1	1.05 (0.81, 1.3)	3.70E-17	321090	0.1	0.99 (0.96, 1.02)	0.41	395066	0.1	1.02 (0.99, 1.06)	0.11	394846
rs7124487	11	2988323	NAP1L4	C	T	0.8	0.53 (0.34, 0.71)	2.40E-08	321090	0.79	1.07 (0.97, 1.19)	0.19	15681	0.79	0.99 (0.91, 1.08)	0.83	15681
rs11601507	11	5701074	TRIM5	A	C	0.07	1.51 (1.21, 1.8)	6.90E-24	321090	0.07	1.03 (0.99, 1.07)	0.12	410785	0.07	1.03 (0.99, 1.07)	0.095	410565
rs11024735	11	18639167	SPTY2D1	T	C	0.74	0.67 (0.5, 0.84)	2.00E-14	321090	0.72	1.01 (0.99, 1.03)	0.37	410806	0.72	1.01 (0.99, 1.03)	0.61	410586
rs174576	11	61603510	FADS2	C	A	0.65	1.29 (1.14, 1.45)	4.80E-59	321090	0.66	1.01 (0.99, 1.03)	0.18	410813	0.66	1.03 (1.01, 1.05)	0.0012	410593
rs59421767	11	66242210	PELI3	A	T	0.75	0.67 (0.49, 0.84)	9.00E-14	321090	0.79	0.99 (0.97, 1.01)	0.37	395066	0.79	1 (0.98, 1.03)	0.79	394846
rs140026975	11	118594669	DDX6	C	A	0.24	0.49 (0.32, 0.67)	3.40E-08	321090	0.25	0.99 (0.97, 1.01)	0.46	395066	0.25	1.01 (0.99, 1.03)	0.55	394846
rs35882350	12	623129	B4GALNT3	G	A	0.26	0.7 (0.53, 0.87)	5.50E-16	321090	0.25	1 (0.98, 1.02)	0.97	395066	0.25	1.02 (1, 1.04)	0.051	394846
rs11054343	12	7687434	-	G	C	0.83	0.59 (0.39, 0.79)	6.80E-09	321090	0.81	0.99 (0.97, 1.02)	0.59	395066	0.81	1.01 (0.99, 1.03)	0.44	394846
rs11047939	12	9061971	PHC1	A	G	0.23	0.75 (0.57, 0.93)	3.10E-16	321090	0.23	0.99 (0.97, 1.02)	0.58	395066	0.23	1.01 (0.99, 1.03)	0.34	394846
rs10877955	12	40421117	SLC2A13	A	G	0.06	0.91 (0.6, 1.22)	6.70E-09	321090	0.06	1.01 (0.97, 1.05)	0.65	410798	0.06	0.99 (0.95, 1.03)	0.51	410578
rs2160994	12	50650057	LIMA1	C	T	0.65	0.68 (0.52, 0.84)	1.80E-17	321090	0.65	1.04 (1.02, 1.06)	0.00016	410788	0.64	0.99 (0.97, 1.01)	0.25	410568
rs10876450	12	53811034	SP1	C	T	0.18	0.66 (0.47, 0.86)	3.50E-11	321090	0.17	1.01 (0.98, 1.03)	0.48	395066	0.17	0.99 (0.96, 1.01)	0.31	394846
rs61754230	12	72179446	RAB21	T	C	0.02	1.81 (1.28, 2.35)	2.90E-11	321090	0.02	0.97 (0.91, 1.03)	0.3	410813	0.02	1.02 (0.96, 1.09)	0.48	410593
rs2220729	12	89929953	GALNT4	A	G	0.35	0.46 (0.3, 0.62)	1.00E-08	321090	0.34	0.99 (0.97, 1.01)	0.2	395066	0.34	0.97 (0.95, 0.99)	0.0021	394846

rs1663564	12	105546172	KIAA1033	G	A	0.1	0.82 (0.57, 1.07)	1.70E-10	321090	0.09	1 (0.97, 1.03)	0.82	410809	0.09	1.02 (0.99, 1.05)	0.18	410589
rs597808	12	111973358	ATXN2	G	A	0.52	0.71 (0.56, 0.86)	1.40E-20	321090	0.51	0.97 (0.96, 0.99)	0.0016	395066	0.51	0.98 (0.96, 1)	0.023	394846
rs1169288	12	121416650	HNF1A	C	A	0.31	1.18 (1.01, 1.34)	8.40E-46	321090	0.32	1.05 (1.03, 1.07)	7.20E-07	395066	0.32	1.01 (0.99, 1.02)	0.58	394846
rs2451321	12	124545435	FAM101A	C	G	0.59	0.5 (0.35, 0.66)	9.00E-11	321090	0.6	1.12 (1.03, 1.22)	0.011	15704	0.6	0.96 (0.89, 1.03)	0.29	15704
rs10846744	12	125312425	SCARB1	C	G	0.14	0.89 (0.68, 1.11)	3.30E-16	321090	0.15	1.05 (1.03, 1.08)	3.00E-05	410750	0.15	1.02 (1, 1.05)	0.063	410530
rs9316496	13	51041880	DLEU1	A	G	0.17	0.57 (0.37, 0.77)	1.70E-08	321090	0.17	1 (0.97, 1.02)	0.7	410759	0.17	1.01 (0.99, 1.03)	0.43	410539
rs9573353	13	74726146	-	T	C	0.52	0.43 (0.28, 0.58)	1.60E-08	321090	0.52	0.92 (0.85, 1)	0.049	15602	0.52	0.94 (0.88, 1.01)	0.12	15602
rs551473284	13	111038325	COL4A2	C	T	0.64	0.5 (0.34, 0.66)	4.10E-10	321090	0.62	0.98 (0.97, 1)	0.083	395066	0.62	1.01 (0.99, 1.03)	0.28	394846
rs11621792	14	24871926	NYNRIN	T	C	0.45	0.82 (0.67, 0.97)	2.80E-26	321090	0.45	0.99 (0.98, 1.01)	0.55	395066	0.45	0.98 (0.96, 1)	0.025	394846
rs7143690	14	70874909	SYNJ2BP	T	C	0.76	0.66 (0.49, 0.84)	1.10E-13	321090	0.73	0.99 (0.97, 1.02)	0.62	410813	0.73	1.01 (0.99, 1.03)	0.56	410593
rs13379043	14	74250126	ELMSAN1	T	C	0.72	0.6 (0.43, 0.77)	5.20E-12	321090	0.71	1 (0.98, 1.02)	0.89	410682	0.71	1 (0.98, 1.01)	0.61	410462
rs17580	14	94847262	SERPINA1	A	T	0.05	1.25 (0.91, 1.6)	1.40E-12	321090	0.04	0.99 (0.95, 1.04)	0.74	410762	0.04	1.01 (0.97, 1.05)	0.71	410542
rs10468017	15	58678512	ALDH1A2	T	C	0.3	0.89 (0.72, 1.05)	2.10E-26	321090	0.29	1.02 (1, 1.04)	0.018	410808	0.29	1.01 (0.99, 1.03)	0.29	410588
rs56369308	15	63792257	USP3	C	T	0.34	0.55 (0.39, 0.71)	6.10E-12	321090	0.35	0.99 (0.97, 1)	0.13	410771	0.35	1 (0.99, 1.02)	0.6	410551
rs12917376	15	75116167	LMAN1L	T	C	0.6	0.49 (0.33, 0.64)	5.10E-10	321090	0.57	0.97 (0.95, 0.99)	0.00082	395066	0.57	1.02 (1, 1.04)	0.03	394846
rs12445804	16	11706100	LITAF	A	G	0.07	1.07 (0.78, 1.36)	3.00E-13	321090	0.06	1.01 (0.97, 1.05)	0.58	395066	0.06	1.03 (0.99, 1.07)	0.15	394846
rs1870293	16	30970941	SETD1A	T	C	0.37	0.51 (0.35, 0.66)	1.30E-10	321090	0.37	1 (0.98, 1.02)	0.88	395066	0.37	1.01 (0.99, 1.02)	0.59	394846
rs821840	16	56993886	CETP	A	G	0.67	1.35 (1.19, 1.51)	6.90E-62	321090	0.68	0.99 (0.97, 1.01)	0.32	410779	0.68	0.99 (0.97, 1.01)	0.36	410559
rs11149612	16	83980965	RP11-505K9.4	C	T	0.54	0.68 (0.53, 0.83)	1.20E-18	321090	0.56	0.98 (0.96, 1)	0.041	410675	0.56	0.99 (0.97, 1.01)	0.28	410455
rs77146052	16	88572019	ZFPM1	T	C	0.02	1.7 (1.13, 2.27)	4.50E-09	321090	0.02	1.06 (0.99, 1.14)	0.1	395066	0.02	1.05 (0.98, 1.13)	0.16	394846
rs55714927	17	7080316	ASGR1	C	T	0.81	1.24 (1.05, 1.43)	2.40E-37	321090	0.82	1.01 (0.99, 1.03)	0.42	410764	0.82	1 (0.97, 1.02)	0.79	410544
rs704	17	26694861	TMEM199; VTN; CTB-96E2.2; SARM1	A	G	0.47	0.6 (0.45, 0.75)	6.00E-15	321090	0.48	1.01 (0.99, 1.02)	0.43	395066	0.48	1.02 (1, 1.03)	0.07	394846
rs8064738	17	66413965	ARSG	G	A	0.76	0.56 (0.39, 0.74)	2.90E-10	321090	0.75	1 (0.98, 1.02)	0.78	395066	0.75	1.01 (0.99, 1.03)	0.36	394846
rs78057960	17	73779075	H3F3B	C	T	0.71	0.5 (0.34, 0.67)	2.10E-09	321090	0.69	1.02 (1, 1.04)	0.1	410766	0.69	1.02 (1, 1.04)	0.039	410546
rs1736182	19	2798686	THOP1	G	T	0.29	0.51 (0.34, 0.67)	1.30E-09	321090	0.3	1.01 (0.99, 1.03)	0.51	395066	0.3	0.99 (0.97, 1.01)	0.25	394846
rs8110695	19	11206530	LDLR	T	A	0.78	3.55 (3.37, 3.74)	3.7e-319	321090	0.79	1.02 (1, 1.05)	0.031	395066	0.79	1 (0.98, 1.03)	0.7	394846
rs4808360	19	15798015	CYP4F12	A	T	0.51	0.53 (0.37, 0.68)	1.10E-11	321090	0.52	1 (0.99, 1.02)	0.62	410715	0.52	1 (0.98, 1.01)	0.75	410495
rs7260450	19	17462094	PLVAP	T	G	0.37	0.46 (0.31, 0.62)	6.60E-09	321090	0.37	1 (0.98, 1.02)	0.91	395066	0.37	0.99 (0.97, 1.01)	0.33	394846
rs12461072	19	18330592	PDE4C	G	C	0.29	0.68 (0.51, 0.84)	7.20E-16	321090	0.28	0.98 (0.96, 1)	0.022	410716	0.29	1.02 (1, 1.04)	0.024	410496
rs56113850	19	41353107	CTC-490E21.12; CYP2A6	C	T	0.58	0.58 (0.42, 0.73)	9.10E-14	321090	0.57	0.97 (0.89, 1.05)	0.45	15684	0.57	0.99 (0.92, 1.06)	0.74	15684
rs111271293	19	45432089	APOC1	T	C	0.9	4.88 (4.63, 5.13)	2e-323	321090	0.9	1.01 (0.98, 1.04)	0.41	395066	0.9	1 (0.97, 1.03)	0.92	394846

rs3764613	19	46896217	PPP5C	A	G	0.43	0.42 (0.27, 0.57)	4.80E-08	321090	0.42	1.01 (0.99, 1.03)	0.21	395066	0.42	1.01 (0.99, 1.03)	0.44	394846
rs679574	19	49206108	FUT2	G	C	0.51	1.25 (1.1, 1.4)	3.30E-60	321090	0.48	0.99 (0.97, 1.01)	0.19	410765	0.48	1.01 (0.99, 1.02)	0.5	410545
rs35081008	19	58662235	ZNF329	C	T	0.85	1.13 (0.92, 1.34)	1.50E-25	321090	0.85	0.99 (0.97, 1.02)	0.65	395066	0.85	1.02 (0.99, 1.04)	0.18	394846
rs55716128	19	59048754	ZBTB45	T	C	0.18	0.56 (0.36, 0.75)	2.60E-08	321090	0.18	1 (0.98, 1.02)	0.85	395066	0.18	0.98 (0.96, 1)	0.084	394846
rs73075609	20	5580789	GPCPD1	T	C	0.03	1.47 (1.01, 1.94)	3.80E-10	321090	0.01	1.07 (0.99, 1.17)	0.097	410734	0.01	1 (0.92, 1.09)	0.96	410514
rs2618567	20	17844492	BANF2	G	T	0.34	1.02 (0.86, 1.18)	5.50E-37	321090	0.36	1.01 (1, 1.03)	0.14	395066	0.36	0.98 (0.97, 1)	0.11	394846
rs11699953	20	25241345	PYGB	G	C	0.48	0.44 (0.29, 0.59)	7.00E-09	321090	0.47	1.02 (1, 1.04)	0.041	395066	0.47	1.01 (0.99, 1.03)	0.27	394846
rs1883711	20	39179822	MAFB	C	G	0.03	4.6 (4.17, 5.04)	1.40E-94	321090	0.03	1.04 (0.97, 1.11)	0.28	395066	0.03	1.05 (0.99, 1.12)	0.13	394846
rs6065904	20	44534651	PLTP	A	G	0.21	0.63 (0.45, 0.81)	9.80E-12	321090	0.22	1 (0.98, 1.02)	0.88	410775	0.22	1.01 (0.99, 1.03)	0.53	410555
rs6127015	20	52540606	BCAS1	C	T	0.54	0.46 (0.31, 0.61)	2.30E-09	321090	0.54	1 (0.99, 1.02)	0.61	395066	0.54	1 (0.99, 1.02)	0.61	394846
rs2427531	20	62362116	ZGPAT; RP4-583P15.15	C	G	0.24	0.5 (0.32, 0.67)	2.20E-08	321090	0.23	1.02 (1, 1.04)	0.039	410767	0.23	0.97 (0.95, 0.99)	0.0013	410547
rs12106385	21	16586682	-	T	A	0.98	1.75 (1.19, 2.3)	7.10E-10	321090	0.98	1 (0.93, 1.09)	0.94	395066	0.98	1.09 (1, 1.18)	0.041	394846
rs138730	22	35696931	TOM1	A	G	0.3	0.54 (0.37, 0.7)	9.80E-11	321090	0.32	1.01 (0.99, 1.03)	0.36	395066	0.32	0.98 (0.96, 1)	0.018	394846

Chr: Chromosome; EA: Effect Allele; NEA: Non-effect Allele; EAF: Effect Allele Frequency.
SNPs excluded in the MR-PRESSO analysis of HFrEF: rs10846744, rs1169288, rs118039278, rs12917376.
SNPs excluded in the MR-PRESSO analysis of HFpEF: rs17185536.

Table S7. GWAS Summary Used for Mendelian Randomization Analysis of Triglycerides.

rsID	Chr	Position (hg19)	Gene	EA	NEA	Triglycerides				HFrEF				HFpEF			
						EAF	Beta (95% CI)	P	N	EAF	OR (95% CI)	P	N	EAF	OR (95% CI)	P	N
rs11206374	1	40048009	PABPC4	A	G	0.23	0.01 (0.01, 0.02)	1.20E-17	321433	0.23	1.01 (0.99, 1.03)	0.6	410723	0.23	1.04 (1.02, 1.06)	0.00042	410503
rs213494	1	54877103	SSBP3	T	C	0.65	0.01 (0.01, 0.01)	1.20E-09	321433	0.63	0.99 (0.97, 1.01)	0.26	395066	0.63	1 (0.99, 1.02)	0.66	394846
rs1690761	1	62955862	DOCK7	T	C	0.65	0.04 (0.04, 0.04)	9.50E-214	321433	0.65	1.02 (1, 1.04)	0.051	410812	0.65	1.01 (0.99, 1.03)	0.22	410592
rs2749732	1	93565123	MTF2	A	G	0.86	0.01 (0.01, 0.02)	4.60E-12	321433	0.86	1.03 (1, 1.06)	0.052	395066	0.86	0.99 (0.97, 1.02)	0.7	394846
rs12138136	1	149844305	HIST2H4 B	T	A	0.91	0.01 (0.01, 0.02)	3.00E-11	321433	0.91	1.03 (0.99, 1.06)	0.11	395066	0.91	1.02 (0.99, 1.05)	0.26	394846
rs9425589	1	172355276	PIGC; DNM3	G	A	0.43	0.01 (0, 0.01)	3.40E-08	321433	0.43	1.01 (0.99, 1.03)	0.3	410747	0.43	1.01 (0.99, 1.03)	0.19	410527
rs3897379	1	219759733	SLC30A1 0	A	G	0.19	0.01 (0.01, 0.02)	1.10E-17	321433	0.2	0.99 (0.97, 1.02)	0.58	410665	0.2	0.99 (0.97, 1.01)	0.45	410445
rs11118610	1	220990086	1-Mar	A	C	0.56	0.01 (0.01, 0.01)	2.40E-12	321433	0.56	0.99 (0.97, 1.01)	0.16	410714	0.56	1.03 (1.02, 1.05)	0.00033	410494
rs11122450	1	230301811	GALNT2	T	G	0.39	0.02 (0.02, 0.03)	1.60E-84	321433	0.4	1.02 (1.01, 1.04)	0.011	410812	0.4	1.01 (0.99, 1.03)	0.42	410592
rs2090032	2	20372547	SDC1	G	C	0.53	0.01 (0.01, 0.01)	4.30E-13	321433	0.53	0.99 (0.97, 1.01)	0.39	395066	0.53	0.99 (0.97, 1.01)	0.29	394846
rs673548	2	21237544	APOB	G	A	0.8	0.04 (0.03, 0.04)	8.70E-134	321433	0.79	1 (0.98, 1.02)	0.89	410812	0.79	1 (0.98, 1.02)	0.82	410592
rs6547692	2	27734972	GCKR	G	A	0.44	0.04 (0.04, 0.05)	3.10E-269	321433	0.47	0.99 (0.98, 1.01)	0.42	410671	0.47	1 (0.98, 1.02)	0.83	410451
rs6739755	2	59330227	-	A	G	0.4	0.01 (0, 0.01)	1.30E-08	321433	0.4	1.03 (1.01, 1.05)	0.0009	395066	0.4	1.02 (1, 1.04)	0.017	394846
rs1009360	2	65276049	CEP68	T	C	0.58	0.01 (0.01, 0.01)	6.10E-17	321433	0.57	0.99 (0.97, 1.01)	0.18	395066	0.57	1.02 (1, 1.04)	0.053	394846
rs6760053	2	111932997	BCL2L11	C	G	0.53	0.01 (0, 0.01)	1.50E-08	321433	0.55	1 (0.98, 1.02)	0.93	395066	0.55	1 (0.98, 1.01)	0.62	394846
rs9752592	2	119757791	MARCO	T	G	0.02	0.03 (0.02, 0.04)	2.30E-09	321433	0.02	0.99 (0.93, 1.07)	0.88	395066	0.02	0.99 (0.92, 1.07)	0.82	394846
rs1128249	2	165528624	COBLL1	G	T	0.61	0.02 (0.02, 0.02)	7.10E-49	321433	0.61	1.09 (1, 1.19)	0.042	15735	0.61	1.01 (0.94, 1.09)	0.75	15735
rs4668314	2	171631258	ERICH2	G	T	0.39	0.01 (0.01, 0.01)	6.40E-11	321433	0.4	1 (0.98, 1.02)	0.87	410744	0.4	1 (0.98, 1.02)	0.89	410524
rs72917544	2	175241566	CIR1	G	A	0.81	0.01 (0.01, 0.01)	2.70E-08	321433	0.83	1 (0.98, 1.03)	0.86	395066	0.83	1.01 (0.99, 1.04)	0.42	394846
rs7645905	3	4758244	ITPR1	T	G	0.51	0.01 (0, 0.01)	5.90E-09	321433	0.55	0.99 (0.97, 1.01)	0.43	395066	0.55	1 (0.99, 1.02)	0.62	394846
rs1899951	3	12394840	PPARG	C	T	0.88	0.02 (0.01, 0.02)	1.20E-15	321433	0.87	1.01 (0.99, 1.04)	0.31	410813	0.87	0.99 (0.96, 1.01)	0.31	410593
rs17052058	3	52344680	DNAH1	A	G	0.82	0.02 (0.01, 0.02)	2.40E-23	321433	0.83	0.99 (0.96, 1.01)	0.24	410733	0.83	1 (0.98, 1.02)	0.91	410513
rs7653249	3	136005792	PCCB	G	C	0.75	0.01 (0.01, 0.02)	2.60E-21	321433	0.75	1.03 (1, 1.05)	0.016	395066	0.75	1.04 (1.02, 1.07)	5.80E-05	394846
rs9653945	3	142660706	PAQR9	G	A	0.65	0.01 (0, 0.01)	7.60E-09	321433	0.66	1.01 (0.99, 1.02)	0.57	395066	0.66	1.01 (0.99, 1.03)	0.17	394846
rs5402	3	170727739	SLC2A2	A	T	0.12	0.01 (0.01, 0.02)	7.80E-11	321433	0.13	1.03 (1, 1.05)	0.043	410814	0.13	1.02 (0.99, 1.04)	0.17	410594
rs79287178	3	172294500	TNFSF10	A	G	0.03	0.03 (0.02, 0.04)	6.90E-17	321433	0.03	0.97 (0.91, 1.03)	0.37	395066	0.03	1.01 (0.95, 1.08)	0.64	394846
rs7631606	3	187628006	-	T	G	0.73	0.01 (0.01, 0.01)	1.10E-09	321433	0.74	1.01 (0.99, 1.03)	0.23	410782	0.74	1.02 (1, 1.05)	0.019	410562
rs7700107	4	17880416	LCORL	C	A	0.14	0.01 (0.01, 0.02)	9.50E-12	321433	0.14	1.02 (1, 1.05)	0.11	410781	0.14	1 (0.97, 1.03)	0.99	410561
rs12513202	4	71894563	DCK	T	C	0.6	0.01 (0.01, 0.01)	4.90E-09	321433	0.6	1.01 (0.99, 1.03)	0.23	410711	0.6	0.99 (0.97, 1.01)	0.31	410491
rs13146355	4	77412140	SHROO M3	A	G	0.46	0.01 (0.01, 0.01)	9.70E-11	321433	0.44	0.97 (0.95, 0.99)	0.00049	410813	0.45	0.98 (0.96, 0.99)	0.0069	410593

rs1471251	4	87976359	AFF1	T	A	0.4	0.02 (0.02, 0.02)	4.00E-50	321433	0.39	0.99 (0.97, 1.01)	0.42	395066	0.39	0.99 (0.97, 1)	0.13	394846
rs3822072	4	89741269	FAM13A	A	G	0.45	0.01 (0.01, 0.01)	1.30E-11	321433	0.46	1.01 (0.99, 1.03)	0.2	410809	0.46	1.01 (0.99, 1.03)	0.36	410589
rs2035816	4	100508556	MTTP	A	G	0.92	0.01 (0.01, 0.02)	2.50E-10	321433	0.91	1 (0.97, 1.03)	0.95	410813	0.91	1.01 (0.98, 1.05)	0.37	410593
rs35225200	4	103146888	SLC39A8	C	A	0.08	0.02 (0.01, 0.02)	4.90E-15	321433	0.09	1.06 (1.03, 1.1)	0.00019	395066	0.09	1.01 (0.98, 1.04)	0.58	394846
rs7735249	5	53310139	ARL15	G	C	0.11	0.01 (0.01, 0.02)	8.40E-11	321433	0.11	1.01 (0.98, 1.04)	0.45	410743	0.11	1 (0.97, 1.03)	0.98	410523
rs4976033	5	67714246	-	G	A	0.4	0.01 (0.01, 0.01)	2.30E-15	321433	0.41	1.01 (0.99, 1.02)	0.55	410693	0.41	0.99 (0.98, 1.01)	0.52	410473
rs7715456	5	78627953	JMY	C	T	0.57	0.01 (0.01, 0.01)	1.60E-09	321433	0.56	1.05 (0.97, 1.14)	0.25	15639	0.56	0.98 (0.91, 1.06)	0.64	15639
rs34580448	5	82810884	VCAN	T	C	0.96	0.02 (0.01, 0.02)	2.50E-08	321433	0.96	1.01 (0.97, 1.05)	0.64	410766	0.96	0.99 (0.95, 1.04)	0.8	410546
rs10519336	5	112480104	MCC	A	G	0.26	0.01 (0.01, 0.01)	1.40E-08	321433	0.25	1.01 (0.99, 1.03)	0.22	410729	0.25	0.99 (0.97, 1.01)	0.43	410509
rs154735	5	131437532	-	A	G	0.06	0.01 (0.01, 0.02)	2.10E-08	321433	0.07	0.99 (0.96, 1.03)	0.68	410810	0.07	1.01 (0.98, 1.05)	0.4	410590
rs72801474	5	132444128	HSPA4	G	A	0.91	0.02 (0.01, 0.02)	6.70E-13	321433	0.92	1.08 (1.05, 1.12)	1.20E-06	395066	0.92	1.01 (0.97, 1.04)	0.71	394846
rs112424890	5	140933792	DIAPH1	T	C	0.18	0.01 (0.01, 0.01)	6.60E-09	321433	0.16	0.99 (0.97, 1.02)	0.51	395066	0.16	1.02 (0.99, 1.04)	0.13	394846
rs4710938	6	20640904	CDKAL1	A	G	0.54	0.01 (0, 0.01)	4.40E-08	321433	0.55	1 (0.98, 1.02)	0.86	410706	0.55	1 (0.98, 1.02)	0.92	410486
rs3131856	6	29607101	GABBR1	T	C	0.87	0.01 (0.01, 0.02)	4.40E-15	321433	0.9	1.05 (1.02, 1.08)	0.0016	410802	0.9	1.01 (0.98, 1.04)	0.48	410582
rs185139895	6	34177853	HMGA1	A	G	0.04	0.02 (0.02, 0.03)	5.00E-13	321433	0.04	1.06 (1.01, 1.11)	0.019	410766	0.04	0.99 (0.94, 1.03)	0.54	410546
rs6458869	6	52630269	GSTA2	C	A	0.36	0.01 (0.01, 0.01)	9.70E-18	321433	0.38	0.98 (0.96, 0.99)	0.012	395066	0.38	1 (0.98, 1.02)	0.95	394846
rs11752394	6	109189664	ARMC2	G	C	0.76	0.01 (0.01, 0.01)	2.50E-10	321433	0.75	1.01 (0.99, 1.03)	0.5	410728	0.75	1 (0.98, 1.02)	0.89	410508
rs6916318	6	127435106	RSPO3	T	A	0.53	0.01 (0.01, 0.02)	1.90E-26	321433	0.51	0.99 (0.98, 1.01)	0.53	410731	0.51	1.01 (0.99, 1.03)	0.35	410511
rs212772	6	133803011	EYA4	G	C	0.46	0.01 (0.01, 0.01)	2.80E-10	321433	0.43	1 (0.98, 1.02)	0.82	395066	0.43	0.98 (0.96, 1)	0.025	394846
rs632057	6	139834012	-	T	G	0.37	0.02 (0.01, 0.02)	2.90E-32	321433	0.37	0.99 (0.97, 1.01)	0.31	410813	0.37	0.99 (0.98, 1.01)	0.52	410593
rs140570886	6	161013013	LPA	T	C	0.98	0.06 (0.05, 0.07)	1.50E-34	321433	0.98	0.82 (0.76, 0.88)	9.00E-08	395066	0.98	0.9 (0.84, 0.98)	0.01	394846
rs73029270	6	164123095	-	A	G	0.87	0.01 (0.01, 0.01)	3.50E-09	321433	0.87	1 (0.97, 1.02)	0.75	395066	0.87	1 (0.97, 1.02)	0.75	394846
rs852392	7	5572132	ACTB	A	G	0.22	0.01 (0.01, 0.01)	4.70E-08	321433	0.22	1.01 (0.99, 1.03)	0.55	395066	0.22	1.02 (1, 1.04)	0.076	394846
rs38205	7	15913588	-	A	C	0.38	0.01 (0.01, 0.01)	7.10E-10	321433	0.39	1.02 (1, 1.03)	0.1	395066	0.39	1.01 (0.99, 1.03)	0.2	394846
rs4410790	7	17284577	-	C	T	0.63	0.01 (0.01, 0.01)	1.20E-10	321433	0.62	1.01 (0.99, 1.02)	0.47	410791	0.62	1.02 (1, 1.04)	0.044	410571
rs2070971	7	44197583	GCK	T	G	0.14	0.01 (0.01, 0.02)	1.30E-11	321433	0.14	1.03 (1, 1.05)	0.041	395066	0.14	1.02 (0.99, 1.04)	0.24	394846
rs13234131	7	73025975	MLXIPL	A	G	0.87	0.07 (0.06, 0.07)	3.30E-274	321433	0.88	1 (0.97, 1.02)	0.86	410809	0.88	0.99 (0.97, 1.02)	0.56	410589
rs6465120	7	76036364	SRCRB4D; ZP3	A	G	0.51	0.01 (0, 0.01)	2.70E-09	321433	0.5	1 (0.99, 1.02)	0.72	395066	0.5	1.02 (1, 1.03)	0.098	394846
rs41749	7	116446882	CAPZA2	C	A	0.55	0.01 (0.01, 0.01)	1.30E-09	321433	0.56	1.01 (0.99, 1.03)	0.4	395066	0.56	1.02 (1.01, 1.04)	0.013	394846
rs55700285	7	150209636	GIMAP7	T	G	0.23	0.01 (0.01, 0.01)	1.20E-09	321433	0.23	0.98 (0.96, 1)	0.1	410726	0.23	0.98 (0.96, 1)	0.061	410506
rs4500049	8	11521079	GATA4	A	T	0.47	0.01 (0.01, 0.02)	2.60E-28	321433	0.47	1.02 (1, 1.04)	0.016	410785	0.47	0.99 (0.97, 1.01)	0.19	410565
rs399485	8	17912752	ASAH1	T	A	0.72	0.01 (0.01, 0.01)	3.50E-09	321433	0.71	1.03 (1.01, 1.05)	0.0065	395066	0.71	1 (0.98, 1.02)	0.98	394846
rs4921913	8	18272377	NAT2	C	T	0.22	0.02 (0.02, 0.02)	1.30E-40	321433	0.23	1.02 (1, 1.04)	0.038	410799	0.23	1.02 (1, 1.04)	0.075	410579
rs10105606	8	19827848	LPL	C	A	0.69	0.05 (0.05, 0.05)	1.00E-296	321433	0.68	1 (0.98, 1.02)	0.94	410811	0.68	1.02 (1, 1.04)	0.017	410591

rs11783984	8	20581264	-	G	C	0.19	0.01 (0.01, 0.01)	3.20E-10	321433	0.18	0.98 (0.96, 1)	0.096	395066	0.18	1 (0.98, 1.02)	0.91	394846
rs2081687	8	59388565	CYP7A1	T	C	0.34	0.01 (0.01, 0.02)	4.90E-23	321433	0.34	0.99 (0.97, 1.01)	0.4	410813	0.34	1.02 (1, 1.04)	0.053	410593
rs13269725	8	72459889	RP11-1102P16.1	G	A	0.08	0.02 (0.01, 0.02)	5.50E-17	321433	0.07	1 (0.96, 1.03)	0.85	395066	0.07	0.98 (0.95, 1.02)	0.28	394846
rs3802177	8	118185025	SLC30A8	G	A	0.69	0.01 (0, 0.01)	2.20E-08	321433	0.69	1.02 (1, 1.04)	0.092	410807	0.69	1.01 (0.99, 1.03)	0.38	410587
rs112875651	8	126506694	TRIB1	G	A	0.61	0.05 (0.05, 0.05)	3.10E-301	321433	0.61	1.03 (1.01, 1.05)	0.0037	395066	0.61	1.01 (0.99, 1.02)	0.58	394846
rs1561928	8	129568061	-	G	A	0.88	0.01 (0.01, 0.01)	3.40E-08	321433	0.87	1.02 (0.99, 1.05)	0.19	410795	0.87	1 (0.97, 1.03)	0.9	410575
rs7855395	9	13676484	-	A	G	0.43	0.01 (0, 0.01)	1.70E-08	321433	0.44	0.99 (0.97, 1.01)	0.27	410662	0.44	0.97 (0.96, 0.99)	0.0043	410442
rs296884	9	86582923	HNRNPK	G	T	0.74	0.01 (0.01, 0.01)	5.40E-17	321433	0.74	0.99 (0.97, 1.01)	0.39	395066	0.74	1 (0.98, 1.02)	0.76	394846
rs12236183	9	92169473	GADD45G	T	C	0.32	0.01 (0.01, 0.01)	1.50E-11	321433	0.33	1.03 (1.01, 1.05)	0.012	395066	0.33	1.01 (0.99, 1.03)	0.47	394846
rs1800978	9	107665978	ABCA1	C	G	0.88	0.01 (0.01, 0.02)	9.30E-12	321433	0.87	1.02 (0.9, 1.15)	0.82	15633	0.87	0.94 (0.84, 1.04)	0.24	15633
rs140107293	10	5267191	AKR1C4	A	G	0.84	0.01 (0.01, 0.02)	2.70E-12	321433	0.85	0.98 (0.96, 1)	0.1	410787	0.85	0.98 (0.95, 1)	0.06	410567
rs1171616	10	61468589	SLC16A9	T	G	0.77	0.01 (0.01, 0.01)	5.00E-11	321433	0.76	1.02 (1, 1.04)	0.082	410789	0.76	1.02 (1, 1.04)	0.096	410569
rs7896783	10	65162153	JMJD1C	G	A	0.53	0.02 (0.02, 0.02)	3.00E-47	321433	0.53	0.99 (0.97, 1)	0.11	410812	0.53	0.99 (0.98, 1.01)	0.42	410592
rs4746297	10	77211912	ZNF503	A	G	0.15	0.01 (0.01, 0.01)	1.50E-08	321433	0.17	1 (0.97, 1.02)	0.73	410747	0.17	1 (0.97, 1.02)	0.9	410527
rs563296	10	99772404	CRTAC1	A	G	0.56	0.01 (0.01, 0.01)	2.00E-12	321433	0.55	1.03 (0.95, 1.12)	0.46	15716	0.55	0.98 (0.91, 1.05)	0.6	15716
rs10883451	10	101924418	ERLIN1	C	T	0.5	0.01 (0, 0.01)	2.80E-08	321433	0.47	0.99 (0.98, 1.01)	0.42	395066	0.47	0.99 (0.97, 1.01)	0.37	394846
rs75398587	10	103946480	NOLC1	C	G	0.93	0.01 (0.01, 0.02)	8.30E-09	321433	0.94	1.02 (0.98, 1.05)	0.39	395066	0.94	1.03 (0.99, 1.07)	0.1	394846
rs74563318	10	114124977	ACSL5	C	A	0.96	0.03 (0.02, 0.03)	1.10E-14	321433	0.97	1.03 (0.97, 1.08)	0.34	395066	0.97	1.03 (0.98, 1.09)	0.22	394846
rs2773469	10	115798895	ADRB1	A	G	0.26	0.01 (0.01, 0.01)	1.10E-13	321433	0.29	1 (0.98, 1.02)	0.67	395066	0.28	0.99 (0.97, 1.01)	0.56	394846
rs11042906	11	10665235	MRVI1	C	T	0.71	0.01 (0, 0.01)	4.80E-08	321433	0.72	1.01 (0.99, 1.03)	0.15	395066	0.72	1.01 (0.99, 1.03)	0.54	394846
rs6486122	11	13361524	ARNTL	T	C	0.69	0.01 (0.01, 0.01)	2.80E-16	321433	0.68	1.01 (1, 1.03)	0.14	410801	0.68	1.03 (1.01, 1.04)	0.0083	410581
rs61885960	11	14819828	PDE3B	T	A	0.94	0.02 (0.01, 0.02)	2.00E-09	321433	0.96	0.95 (0.9, 1)	0.032	395066	0.96	0.97 (0.92, 1.02)	0.21	394846
rs11030107	11	27694835	BDNF	G	A	0.26	0.01 (0.01, 0.01)	1.40E-09	321433	0.25	1.02 (1, 1.04)	0.021	410789	0.25	1.02 (1, 1.04)	0.021	410569
rs77756595	11	45875161	CRY2	A	G	0.96	0.02 (0.01, 0.03)	1.50E-09	321433	0.97	1.01 (0.95, 1.06)	0.82	395066	0.97	1.02 (0.97, 1.08)	0.4	394846
rs10838681	11	47275064	NR1H3	G	A	0.73	0.01 (0.01, 0.02)	1.50E-22	321433	0.74	0.97 (0.96, 0.99)	0.011	410814	0.74	1.02 (1, 1.04)	0.056	410594
rs7934157	11	60955332	VPS37C	G	A	0.95	0.02 (0.01, 0.02)	1.40E-08	321433	0.95	1.03 (0.99, 1.07)	0.19	395066	0.95	1.01 (0.97, 1.06)	0.49	394846
rs174574	11	61600342	FADS2	A	C	0.35	0.03 (0.02, 0.03)	2.70E-94	321433	0.35	0.99 (0.97, 1.01)	0.17	410811	0.35	0.97 (0.95, 0.99)	0.0007	410591
rs1801144	11	62381808	ROM1	C	G	0.37	0.01 (0.01, 0.01)	1.30E-10	321433	0.35	0.99 (0.97, 1.01)	0.46	395066	0.35	1 (0.98, 1.02)	0.71	394846
rs678614	11	64799894	RP11-399J13.3; SNX15	A	C	0.28	0.01 (0.01, 0.01)	1.00E-10	321433	0.29	1 (0.98, 1.02)	0.68	410730	0.29	1.01 (0.99, 1.03)	0.29	410510
rs10750766	11	65473798	KAT5	A	C	0.71	0.01 (0.01, 0.01)	5.00E-13	321433	0.7	1 (0.98, 1.02)	0.96	395066	0.7	1.02 (1, 1.04)	0.02	394846
rs11605837	11	68597886	CPT1A	T	G	0.31	0.01 (0.01, 0.01)	2.60E-10	321433	0.33	0.99 (0.97, 1.01)	0.38	410715	0.33	1.01 (0.99, 1.02)	0.56	410495
rs2850245	11	111760738	C11orf1	G	T	0.37	0.01 (0.01, 0.01)	7.30E-11	321433	0.37	1 (0.98, 1.02)	0.77	395066	0.37	1 (0.98, 1.02)	0.99	394846
rs2070665	11	116707684	APOA1	A	G	0.07	0.09 (0.09, 0.1)	6.4e-317	321433	0.09	1.01 (0.97, 1.04)	0.73	410807	0.09	1 (0.97, 1.03)	0.91	410587

rs11045171	12	20470199	PDE3A	A	G	0.8	0.01 (0.01, 0.02)	1.70E-21	321433	0.8	1 (0.97, 1.02)	0.71	395066	0.8	1 (0.97, 1.02)	0.78	394846
rs67981690	12	21343886	SLCO1B1	G	A	0.13	0.02 (0.01, 0.02)	7.00E-21	321433	0.14	1.01 (0.98, 1.04)	0.52	395066	0.14	0.99 (0.97, 1.02)	0.59	394846
rs4760254	12	57766392	R3HDM2	G	C	0.76	0.01 (0.01, 0.02)	2.50E-21	321433	0.77	1.01 (0.99, 1.03)	0.26	410782	0.77	0.97 (0.95, 0.99)	0.0023	410562
rs113439801	12	62838230	MON2	C	T	0.83	0.01 (0.01, 0.01)	9.80E-11	321433	0.83	0.99 (0.97, 1.02)	0.51	395066	0.83	1.02 (0.99, 1.04)	0.21	394846
rs863750	12	124505444	FAM101A	T	C	0.6	0.01 (0.01, 0.02)	7.40E-31	321433	0.59	1.02 (1, 1.03)	0.068	410790	0.59	1 (0.99, 1.02)	0.6	410570
rs11057837	12	125311720	SCARB1	T	C	0.1	0.01 (0.01, 0.02)	6.20E-09	321433	0.09	1.05 (1.01, 1.08)	0.0057	395066	0.09	1.02 (0.99, 1.06)	0.2	394846
rs1928496	13	31012904	HMGB1	T	C	0.74	0.01 (0.01, 0.01)	4.80E-09	321433	0.75	1.02 (1, 1.04)	0.11	410801	0.75	1.01 (0.99, 1.03)	0.26	410581
rs2812208	13	50707087	DLEU1	G	C	0.98	0.03 (0.02, 0.04)	7.60E-11	321433	0.98	0.99 (0.93, 1.06)	0.79	395066	0.98	1.02 (0.96, 1.09)	0.5	394846
rs12880341	14	64236191	-	C	T	0.16	0.01 (0.01, 0.01)	8.80E-09	321433	0.16	1.1 (0.99, 1.22)	0.09	15736	0.16	0.95 (0.87, 1.05)	0.35	15736
rs34245505	15	40397191	BMF	G	C	0.2	0.01 (0.01, 0.01)	9.10E-10	321433	0.19	1.02 (1, 1.05)	0.066	395066	0.19	0.99 (0.96, 1.01)	0.25	394846
rs11636523	15	42051902	MGA	T	C	0.69	0.01 (0.01, 0.01)	3.40E-10	321433	0.7	1.01 (0.99, 1.03)	0.16	410761	0.7	1 (0.98, 1.02)	0.89	410541
rs147233090	15	44028047	PDIA3	T	C	0.02	0.08 (0.07, 0.09)	2.40E-81	321433	0.03	0.97 (0.92, 1.03)	0.32	395066	0.03	0.97 (0.91, 1.03)	0.3	394846
rs261342	15	58731153	ALDH1A2; LIPC	G	C	0.22	0.02 (0.02, 0.02)	1.30E-46	321433	0.23	1.02 (1, 1.05)	0.054	395066	0.23	1.02 (1, 1.04)	0.11	394846
rs12591786	15	60902512	RORA	C	T	0.84	0.01 (0.01, 0.01)	1.20E-08	321433	0.84	0.99 (0.97, 1.02)	0.61	395066	0.84	1 (0.98, 1.03)	0.9	394846
rs12440800	15	61960302	-	T	A	0.25	0.01 (0.01, 0.01)	1.30E-08	321433	0.26	0.99 (0.97, 1.02)	0.6	395066	0.26	0.99 (0.97, 1.01)	0.27	394846
rs11635675	15	63793238	USP3	G	T	0.34	0.01 (0.01, 0.02)	2.80E-23	321433	0.35	0.99 (0.97, 1)	0.13	410796	0.35	1.01 (0.99, 1.02)	0.57	410576
rs4776794	15	66872266	RP11-321F6.1	A	G	0.35	0.01 (0.01, 0.01)	1.60E-11	321433	0.35	1 (0.98, 1.02)	0.98	410806	0.35	1.01 (0.99, 1.03)	0.45	410586
rs2017500	15	99196112	IGF1R	A	G	0.51	0.01 (0, 0.01)	9.80E-09	321433	0.5	1.01 (0.99, 1.03)	0.31	410762	0.5	1.02 (1, 1.04)	0.016	410542
rs8025505	15	102067841	PCSK6	T	C	0.26	0.01 (0.01, 0.01)	5.20E-16	321433	0.26	1.06 (0.97, 1.16)	0.22	15681	0.26	0.99 (0.91, 1.07)	0.82	15681
rs200841050	16	2450570	CCNF	C	T	0.33	0.01 (0, 0.01)	1.70E-08	321433	0.35	0.98 (0.96, 1)	0.029	395066	0.35	0.99 (0.97, 1)	0.13	394846
rs1684608	16	4676852	MGRN1	A	C	0.19	0.01 (0.01, 0.01)	4.80E-09	321433	0.18	0.99 (0.97, 1.02)	0.56	395066	0.18	0.99 (0.97, 1.01)	0.43	394846
rs12929396	16	11774625	TXNDC11	T	C	0.49	0.01 (0, 0.01)	3.10E-09	321433	0.48	1.01 (0.99, 1.02)	0.45	410665	0.48	0.99 (0.97, 1)	0.13	410445
rs12446515	16	56987015	CETP	C	T	0.68	0.02 (0.02, 0.02)	2.00E-40	321433	0.68	0.99 (0.97, 1.01)	0.27	410810	0.68	0.99 (0.97, 1.01)	0.39	410590
rs244418	16	69622762	NFAT5	G	A	0.59	0.01 (0.01, 0.01)	1.40E-14	321433	0.59	1.05 (1.03, 1.07)	8.30E-08	395066	0.59	1.03 (1.02, 1.05)	0.00035	394846
rs2000999	16	72108093	TXNL4B; HPR	A	G	0.19	0.01 (0.01, 0.02)	2.50E-19	321433	0.2	1.03 (1.01, 1.06)	0.0018	410800	0.2	1.02 (1, 1.04)	0.044	410580
rs79311290	16	85150163	FAM92B	G	A	0.11	0.01 (0.01, 0.02)	3.20E-09	321433	0.12	1.03 (1, 1.06)	0.088	395066	0.12	1.03 (0.99, 1.06)	0.097	394846
rs11078597	17	1618363	WDR81	C	T	0.19	0.01 (0.01, 0.01)	1.40E-11	321433	0.19	1.04 (1.01, 1.06)	0.002	410774	0.18	1 (0.97, 1.02)	0.67	410554
rs56030759	17	17482025	PEMT	C	T	0.06	0.02 (0.02, 0.03)	1.70E-17	321433	0.06	0.95 (0.91, 0.98)	0.0028	410671	0.06	1.01 (0.97, 1.04)	0.7	410451
rs71372276	17	18200779	TOP3A	G	C	0.6	0.01 (0.01, 0.01)	1.60E-09	321433	0.61	1 (0.98, 1.02)	0.83	395066	0.61	1 (0.98, 1.02)	0.7	394846
rs241771	17	26592946	AC061975.10	C	T	0.55	0.01 (0, 0.01)	1.00E-08	321433	0.54	0.99 (0.98, 1.01)	0.4	410798	0.54	0.99 (0.97, 1.01)	0.3	410578
rs10775406	17	46197755	SNX11	G	A	0.76	0.01 (0.01, 0.01)	1.50E-13	321433	0.74	1 (0.98, 1.02)	0.78	410799	0.74	0.99 (0.97, 1.01)	0.59	410579
rs11079868	17	47357969	ZNF652	A	G	0.45	0.01 (0.01, 0.01)	4.90E-11	321433	0.46	1.02 (1, 1.04)	0.029	410707	0.46	1.03 (1.01, 1.04)	0.0043	410487
rs1292065	17	57906288	VMP1	C	G	0.29	0.01 (0.01, 0.01)	1.50E-10	321433	0.31	1 (0.98, 1.02)	0.65	395066	0.31	1.01 (1, 1.03)	0.14	394846
rs164011	17	74273165	QRICH2	G	A	0.68	0.01 (0.01, 0.01)	5.70E-10	321433	0.68	1.02 (1, 1.04)	0.11	395066	0.68	1 (0.98, 1.02)	0.84	394846

rs4969179	17	76391454	PGS1	T	G	0.4	0.01 (0.01, 0.01)	5.10E-13	321433	0.39	1.03 (0.95, 1.12)	0.46	15683	0.39	0.96 (0.89, 1.03)	0.25	15683
rs8088001	18	287705	THOC1	T	G	0.93	0.01 (0.01, 0.02)	8.30E-10	321433	0.92	0.99 (0.96, 1.02)	0.47	395066	0.92	1 (0.97, 1.04)	0.78	394846
rs12964689	18	21116998	NPC1	A	G	0.51	0.01 (0.01, 0.01)	9.80E-12	321433	0.52	1.02 (1, 1.04)	0.029	410794	0.52	1.01 (0.99, 1.03)	0.19	410574
rs68033110	18	57914679	-	A	G	0.25	0.01 (0.01, 0.01)	3.00E-08	321433	0.24	1.04 (1.02, 1.06)	0.0003	395066	0.24	1.01 (0.99, 1.03)	0.38	394846
rs28540102	19	4975763	KDM4B	C	T	0.66	0.01 (0.01, 0.01)	1.50E-09	321433	0.64	1.02 (1, 1.04)	0.11	395066	0.64	0.98 (0.97, 1)	0.11	394846
rs2042901	19	7204394	INSR	T	G	0.26	0.01 (0.01, 0.01)	2.80E-16	321433	0.26	1.02 (1, 1.04)	0.11	410733	0.26	1.01 (0.99, 1.03)	0.3	410513
rs62102718	19	33891013	PEPD	T	A	0.29	0.01 (0.01, 0.01)	7.60E-15	321433	0.27	1 (0.98, 1.02)	0.78	395066	0.27	0.99 (0.97, 1.01)	0.45	394846
rs11671010	19	35559474	HPN	C	T	0.18	0.01 (0.01, 0.02)	5.30E-15	321433	0.16	1 (0.98, 1.02)	0.98	410809	0.16	1 (0.98, 1.03)	0.78	410589
rs483082	19	45416178	APOC1	T	G	0.24	0.05 (0.04, 0.05)	1.10E-216	321433	0.23	0.99 (0.96, 1.01)	0.18	395066	0.23	0.98 (0.96, 1)	0.078	394846
rs41275764	19	46857257	PPP5C	G	A	0.69	0.01 (0.01, 0.01)	5.30E-10	321433	0.7	1 (0.98, 1.02)	0.68	395066	0.7	0.99 (0.97, 1.01)	0.19	394846
rs838133	19	49259529	FGF21	A	G	0.45	0.01 (0.01, 0.01)	4.40E-20	321433	0.45	0.99 (0.91, 1.08)	0.87	15596	0.45	0.98 (0.91, 1.06)	0.63	15596
rs142385484	19	50016759	FCGRT	C	T	0.85	0.01 (0.01, 0.01)	1.30E-10	321433	0.85	0.97 (0.95, 1)	0.047	395066	0.85	1.03 (1, 1.06)	0.042	394846
rs12610709	19	56102362	FIZ1	A	G	0.17	0.01 (0.01, 0.02)	2.90E-14	321433	0.16	1.02 (0.99, 1.04)	0.12	395066	0.16	1 (0.98, 1.03)	0.89	394846
rs117113213	20	39165692	-	A	G	0.03	0.03 (0.02, 0.04)	7.00E-17	321433	0.03	1.04 (0.97, 1.1)	0.3	395066	0.03	1.05 (0.98, 1.12)	0.15	394846
rs6066138	20	45594711	EYA2	G	A	0.72	0.01 (0.01, 0.01)	2.10E-13	321433	0.73	0.99 (0.97, 1.01)	0.2	410812	0.73	1 (0.98, 1.02)	0.97	410592
rs7274718	20	56113783	CTCFL	A	G	0.6	0.01 (0.01, 0.01)	1.60E-10	321433	0.59	1.02 (1.01, 1.04)	0.0099	395066	0.59	0.99 (0.98, 1.01)	0.55	394846
rs8126001	20	62711459	OPRL1	C	T	0.51	0.01 (0.01, 0.01)	8.50E-12	321433	0.53	1 (0.98, 1.02)	0.9	395066	0.53	0.99 (0.97, 1.01)	0.22	394846
rs9610329	22	36042986	APOL6	T	C	0.43	0.01 (0.01, 0.01)	4.60E-10	321433	0.43	1.03 (0.94, 1.11)	0.55	15633	0.43	1.03 (0.96, 1.11)	0.37	15633
rs2267373	22	38600542	PLA2G6; MAFF	T	C	0.58	0.01 (0.01, 0.01)	1.90E-19	321433	0.57	0.99 (0.97, 1.01)	0.22	410813	0.57	0.99 (0.97, 1.01)	0.19	410593
rs113658408	22	46646019	CDPF1	G	C	0.1	0.01 (0.01, 0.02)	4.60E-09	321433	0.1	1.03 (1, 1.06)	0.07	410798	0.1	1.01 (0.98, 1.04)	0.38	410578

Chr: Chromosome; EA: Effect Allele; NEA: Non-effect Allele; EAF: Effect Allele Frequency.
SNPs excluded in the MR-PRESSO analysis of HFrEF: rs13146355, rs140570886, rs244418, rs72801474.
SNPs excluded in the MR-PRESSO analysis of HFpEF: rs11206374, rs174574, rs7653249.

VA Million Veteran Program:
Core Acknowledgement for Publications
February 2023

MVP Program Office

- Sumitra Muralidhar, Ph.D., Program Director
  US Department of Veterans Affairs, 810 Vermont Avenue NW, Washington, DC 20420
- Jennifer Moser, Ph.D., Associate Director, Scientific Programs
  US Department of Veterans Affairs, 810 Vermont Avenue NW, Washington, DC 20420
- Jennifer E. Deen, B.S., Associate Director, Cohort & Public Relations
  US Department of Veterans Affairs, 810 Vermont Avenue NW, Washington, DC 20420

MVP Executive Committee

- Co-Chair: Philip S. Tsao, Ph.D.
  VA Palo Alto Health Care System, 3801 Miranda Avenue, Palo Alto, CA 94304
- Co-Chair: Sumitra Muralidhar, Ph.D.
  US Department of Veterans Affairs, 810 Vermont Avenue NW, Washington, DC 20420
- J. Michael Gaziano, M.D., M.P.H.
  VA Boston Healthcare System, 150 S. Huntington Avenue, Boston, MA 02130
- Elizabeth Hauser, Ph.D.
  Durham VA Medical Center, 508 Fulton Street, Durham, NC 27705
- Amy Kilbourne, Ph.D., M.P.H.
  VA HSR&D, 2215 Fuller Road, Ann Arbor, MI 48105
- Shiuh-Wen Luoh, M.D., Ph.D.
  VA Portland Health Care System, 3710 SW US Veterans Hospital Rd, Portland, OR 97239
- Michael Matheny, M.D., M.S., M.P.H.
  VA Tennessee Valley Healthcare System, 1310 24th Ave. South, Nashville, TN 37212
- Dave Oslin, M.D.
  Philadelphia VA Medical Center, 3900 Woodland Avenue, Philadelphia, PA 19104

MVP Co-Principal Investigators

- J. Michael Gaziano, M.D., M.P.H.
  VA Boston Healthcare System, 150 S. Huntington Avenue, Boston, MA 02130
- Philip S. Tsao, Ph.D.
  VA Palo Alto Health Care System, 3801 Miranda Avenue, Palo Alto, CA 94304

MVP Core Operations

- Lori Churby, B.S., Director, MVP Regulatory Affairs
  VA Palo Alto Health Care System, 3801 Miranda Avenue, Palo Alto, CA 94304
- Stacey B. Whitbourne, Ph.D., Director, MVP Cohort Management
  VA Boston Healthcare System, 150 S. Huntington Avenue, Boston, MA 02130
- Jessica V. Brewer, M.P.H., Director, MVP Recruitment & Enrollment

VA Boston Healthcare System, 150 S. Huntington Avenue, Boston, MA 02130
- Shahpoor (Alex) Shayan, M.S., Director, MVP Recruitment and Enrollment Informatics
  VA Boston Healthcare System, 150 S. Huntington Avenue, Boston, MA 02130
- Luis E. Selva, Ph.D., Executive Director, MVP Biorepositories
  VA Boston Healthcare System, 150 S. Huntington Avenue, Boston, MA 02130
- Saiju Pyarajan Ph.D., Director, Data and Computational Sciences
  VA Boston Healthcare System, 150 S. Huntington Avenue, Boston, MA 02130
- Kelly Cho, M.P.H, Ph.D., Director, MVP Phenomics Data Core
  VA Boston Healthcare System, 150 S. Huntington Avenue, Boston, MA 02130
- Scott L. DuVall, Ph.D., Director, VA Informatics and Computing Infrastructure (VINCI)
  VA Salt Lake City Health Care System, 500 Foothill Drive, Salt Lake City, UT 84148
- Mary T. Brophy M.D., M.P.H., Director, VA Central Biorepository
  VA Boston Healthcare System, 150 S. Huntington Avenue, Boston, MA 02130
- MVP Coordinating Centers
  - MVP Coordinating Center, Boston - J. Michael Gaziano, M.D., M.P.H.
    VA Boston Healthcare System, 150 S. Huntington Avenue, Boston, MA 02130
  - MVP Coordinating Center, Palo Alto – Philip S. Tsao, Ph.D.
    VA Palo Alto Health Care System, 3801 Miranda Avenue, Palo Alto, CA 94304
  - MVP Information Center, Canandaigua – Brady Stephens, M.S.
    Canandaigua VA Medical Center, 400 Fort Hill Avenue, Canandaigua, NY 14424
  - Cooperative Studies Program Clinical Research Pharmacy Coordinating Center, Albuquerque – Todd Connor, Pharm.D.; Dean P. Argyres, B.S., M.S.
    New Mexico VA Health Care System, 1501 San Pedro Drive SE, Albuquerque, NM 87108

MVP Publications and Presentations Committee

- Co-Chair: Themistocles L. Assimes, M.D., Ph. D
  VA Palo Alto Health Care System, 3801 Miranda Avenue, Palo Alto, CA 94304
- Co-Chair: Adriana Hung, M.D.; M.P.H
  VA Tennessee Valley Healthcare System, 1310 24th Ave. South, Nashville, TN 37212
- Co-Chair: Henry Kranzler, M.D.
  Philadelphia VA Medical Center, 3900 Woodland Avenue, Philadelphia, PA 19104

MVP Local Site Investigators

- Samuel Aguayo, M.D., Phoenix VA Health Care System
  650 E. Indian School Road, Phoenix, AZ 85012
- Sunil Ahuja, M.D., South Texas Veterans Health Care System
  7400 Merton Minter Boulevard, San Antonio, TX 78229
- Kathrina Alexander, M.D., Veterans Health Care System of the Ozarks
  1100 North College Avenue, Fayetteville, AR 72703
- Xiao M. Androulakis, M.D., Columbia VA Health Care System
  6439 Garners Ferry Road, Columbia, SC 29209
- Prakash Balasubramanian, M.D., William S. Middleton Memorial Veterans Hospital
  2500 Overlook Terrace, Madison, WI 53705

- Zuhair Ballas, M.D., Iowa City VA Health Care System
  601 Highway 6 West, Iowa City, IA 52246-2208
- Jean Beckham, Ph.D., Durham VA Medical Center
  508 Fulton Street, Durham, NC 27705
- Sujata Bhushan, M.D., VA North Texas Health Care System
  4500 S. Lancaster Road, Dallas, TX 75216
- Edward Boyko, M.D., VA Puget Sound Health Care System
  1660 S. Columbian Way, Seattle, WA 98108-1597
- David Cohen, M.D., Portland VA Medical Center
  3710 SW U.S. Veterans Hospital Road, Portland, OR 97239
- Louis Dellitalia, M.D., Birmingham VA Medical Center
  700 S. 19th Street, Birmingham AL 35233
- L. Christine Faulk, M.D., Robert J. Dole VA Medical Center
  5500 East Kellogg Drive, Wichita, KS 67218-1607
- Joseph Fayad, M.D., VA Southern Nevada Healthcare System
  6900 North Pecos Road, North Las Vegas, NV 89086
- Daryl Fujii, Ph.D., VA Pacific Islands Health Care System
  459 Patterson Rd, Honolulu, HI 96819
- Saib Gappy, M.D., John D. Dingell VA Medical Center
  4646 John R Street, Detroit, MI 48201
- Frank Gesek, Ph.D., White River Junction VA Medical Center
  163 Veterans Drive, White River Junction, VT 05009
- Jennifer Greco, M.D., Sioux Falls VA Health Care System
  2501 W 22nd Street, Sioux Falls, SD 57105
- Michael Godschalk, M.D., Richmond VA Medical Center
  1201 Broad Rock Blvd., Richmond, VA 23249
- Todd W. Gress, M.D., Ph.D., Hershel “Woody” Williams VA Medical Center
  1540 Spring Valley Drive, Huntington, WV 25704
- Samir Gupta, M.D., M.S.C.S., VA San Diego Healthcare System
  3350 La Jolla Village Drive, San Diego, CA 92161
- Salvador Gutierrez, M.D., Edward Hines, Jr. VA Medical Center
  5000 South 5th Avenue, Hines, IL 60141
- John Harley, M.D., Ph.D., Cincinnati VA Medical Center
  3200 Vine Street, Cincinnati, OH 45220
- Kimberly Hammer, Ph.D., Fargo VA Health Care System
  2101 N. Elm, Fargo, ND 58102
- Mark Hamner, M.D., Ralph H. Johnson VA Medical Center
  109 Bee Street, Mental Health Research, Charleston, SC 29401
- Adriana Hung, M.D., M.P.H., VA Tennessee Valley Healthcare System
  1310 24th Avenue, South Nashville, TN 37212
- Robin Hurley, M.D., W.G. (Bill) Hefner VA Medical Center
  1601 Brenner Ave, Salisbury, NC 28144
- Pran Iruvanti, D.O., Ph.D., Hampton VA Medical Center
  100 Emancipation Drive, Hampton, VA 23667
- Frank Jacono, M.D., VA Northeast Ohio Healthcare System
  10701 East Boulevard, Cleveland, OH 44106

- Darshana Jhala, M.D., Philadelphia VA Medical Center
  3900 Woodland Avenue, Philadelphia, PA 19104
- Scott Kinlay, M.B.B.S., Ph.D., VA Boston Healthcare System
  150 S. Huntington Avenue, Boston, MA 02130
- Jon Klein, M.D., Ph.D., Louisville VA Medical Center
  800 Zorn Avenue, Louisville, KY 40206
- Michael Landry, Ph.D., Southeast Louisiana Veterans Health Care System
  2400 Canal Street, New Orleans, LA 70119
- Peter Liang, M.D., M.P.H., VA New York Harbor Healthcare System
  423 East 23rd Street, New York, NY 10010
- Suthat Liangpunsakul, M.D., M.P.H., Richard Roudebush VA Medical Center
  1481 West 10th Street, Indianapolis, IN 46202
- Jack Lichy, M.D., Ph.D., Washington DC VA Medical Center
  50 Irving St, Washington, D. C. 20422
- C. Scott Mahan, M.D., Charles George VA Medical Center
  1100 Tunnel Road, Asheville, NC 28805
- Ronnie Marrache, M.D., VA Maine Healthcare System
  1 VA Center, Augusta, ME 04330
- Stephen Mastorides, M.D., James A. Haley Veterans' Hospital
  13000 Bruce B. Downs Blvd, Tampa, FL 33612
- Elisabeth Mates M.D., Ph.D., VA Sierra Nevada Health Care System
  975 Kirman Avenue, Reno, NV 89502
- Kristin Mattocks, Ph.D., M.P.H., Central Western Massachusetts Healthcare System
  421 North Main Street, Leeds, MA 01053
- Paul Meyer, M.D., Ph.D., Southern Arizona VA Health Care System
  3601 S 6th Avenue, Tucson, AZ 85723
- Jonathan Moorman, M.D., Ph.D., James H. Quillen VA Medical Center
  Corner of Lamont & Veterans Way, Mountain Home, TN 37684
- Timothy Morgan, M.D., VA Long Beach Healthcare System
  5901 East 7th Street Long Beach, CA 90822
- Maureen Murdoch, M.D., M.P.H., Minneapolis VA Health Care System
  One Veterans Drive, Minneapolis, MN 55417
- James Norton, Ph.D., VA Health Care Upstate New York
  113 Holland Avenue, Albany, NY 12208
- Olaoluwa Okusaga, M.D., Michael E. DeBakey VA Medical Center
  2002 Holcombe Blvd, Houston, TX 77030
- Kris Ann Oursler, M.D., Salem VA Medical Center
  1970 Roanoke Blvd, Salem, VA 24153
- Ana Palacio, M.D., M.P.H., Miami VA Health Care System
  1201 NW 16th Street, 11 GRC, Miami FL 33125
- Samuel Poon, M.D., Manchester VA Medical Center
  718 Smyth Road, Manchester, NH 03104
- Emily Potter, Pharm.D., VA Eastern Kansas Health Care System
  4101 S 4th Street Trafficway, Leavenworth, KS 66048
- Michael Rauchman, M.D., St. Louis VA Health Care System
  915 North Grand Blvd, St. Louis, MO 63106

- Richard Servatius, Ph.D., Syracuse VA Medical Center
  800 Irving Avenue, Syracuse, NY 13210
- Satish Sharma, M.D., Providence VA Medical Center
  830 Chalkstone Avenue, Providence, RI 02908
- River Smith, Ph.D., Eastern Oklahoma VA Health Care System
  1011 Honor Heights Drive, Muskogee, OK 74401
- Peruvemba Sriram, M.D., N. FL/S. GA Veterans Health System
  1601 SW Archer Road, Gainesville, FL 32608
- Patrick Strollo, Jr., M.D., VA Pittsburgh Health Care System
  University Drive, Pittsburgh, PA 15240
- Neeraj Tandon, M.D., Overton Brooks VA Medical Center
  510 East Stoner Ave, Shreveport, LA 71101
- Philip Tsao, Ph.D., VA Palo Alto Health Care System
  3801 Miranda Avenue, Palo Alto, CA 94304-1290
- Gerardo Villareal, M.D., New Mexico VA Health Care System
  1501 San Pedro Drive, S.E. Albuquerque, NM 87108
- Agnes Wallbom, M.D., M.S., VA Greater Los Angeles Health Care System
  11301 Wilshire Blvd, Los Angeles, CA 90073
- Jessica Walsh, M.D., VA Salt Lake City Health Care System
  500 Foothill Drive, Salt Lake City, UT 84148
- John Wells, Ph.D., Edith Nourse Rogers Memorial Veterans Hospital
  200 Springs Road, Bedford, MA 01730
- Jeffrey Whittle, M.D., M.P.H., Clement J. Zablocki VA Medical Center
  5000 West National Avenue, Milwaukee, WI 53295
- Mary Whooley, M.D., San Francisco VA Health Care System
  4150 Clement Street, San Francisco, CA 94121
- Allison E. Williams, N.D., Ph.D., R.N, Bay Pines VA Healthcare System
  10,000 Bay Pines Blvd Bay Pines, FL 33744
- Peter Wilson, M.D., Atlanta VA Medical Center
  1670 Clairmont Road, Decatur, GA 30033
- Junzhe Xu, M.D., VA Western New York Healthcare System
  3495 Bailey Avenue, Buffalo, NY 14215-1199
- Shing Shing Yeh, Ph.D., M.D., Northport VA Medical Center
  79 Middleville Road, Northport, NY 11768
